# Reformulating Small Molecules for Cardiovascular Disease Immune Intervention: Low-Dose Combined Vitamin D/Dexamethasone Promotes IL-10 Production and Atheroprotection in Dyslipidemic Mice

**DOI:** 10.3389/fimmu.2020.00743

**Published:** 2020-04-24

**Authors:** Laura Ospina-Quintero, Julio C. Jaramillo, Jorge H. Tabares-Guevara, José R. Ramírez-Pineda

**Affiliations:** Grupo Inmunomodulación (GIM), Instituto de Investigaciones Médicas, Facultad de Medicina, Corporación Académica para el Estudio de Patologías Tropicales (CAEPT), Universidad de Antioquia, Medellin, Colombia

**Keywords:** atherosclerosis, immune intervention, Vitamin D3, Dexamethasone, interleukin-10, immunoregulatory cells

## Abstract

The targeting of proinflammatory pathways has a prophylactic and therapeutic potential on atherosclerotic cardiovascular diseases (CVD). An alternative/complementary strategy is the promotion of endogenous atheroprotective mechanisms that are impaired during atherosclerosis progression, such as the activity of tolerogenic dendritic cells (tolDC) and regulatory T cells (Treg). There is a need to develop novel low cost, safe and effective tolDC/Treg-inducing formulations that are atheroprotective and that can be of easy translation into clinical settings. We found that apolipoprotein E-deficient (ApoE^–/–^) mice treated with a low-dose combined formulation of Vitamin D and Dexamethasone (VitD/Dexa), delivered repetitively and subcutaneously (sc) promoted interleukin-10 (IL-10) production by dendritic cells and other antigen presenting cells in the lymph nodes draining the site of injection and the spleens. Expectedly, the treatment also increased the numbers of IL-10-producing CD4^+^ T cells. Concomitantly, the frequency of IFNγ-producing CD4^+^ and CD8^+^ T cells in the spleen, and the IFNγ response of splenocytes to polyclonal stimulation *ex vivo* were lower after VitD/Dexa treatment, indicating a reduced proatherogenic Th1 response. Interestingly, VitD/Dexa-treated mice had smaller atherosclerotic lesions, with reduced lipid content and lower inflammatory infiltrate of macrophages and T cells in the aortic root. No hypolipidemic or antioxidant effect could be detected, suggesting that a dominantly immunomodulatory mechanism of atheroprotection was engaged under the low-dose sc VitD/Dexa conditions used. Finally, no evidence of clinical, biochemical or immune toxicity was observed in treated ApoE^–/–^ mice and, most importantly, C57BL/6 mice latently infected with *Leishmania* parasites and treated with an identical VitD/Dexa dose/scheme showed no clinical or microbiological signs of disease reactivation, suggesting the absence of general immunosuppression. Altogether, these results indicate that a non-toxic, non-immunosuppressive, low-dose of VitD/Dexa, administered subcutaneously and repetitively, exerts atheroprotective effects in dyslipidemic mice, apparently due to the induction of an IL-10-producing network of lymphoid and myeloid immune cells. These well known, widely available, and inexpensive small molecules can be easily co-formulated into a simple and accessible agent with a potential use as a prophylactic or therapeutic immune intervention for CVD and other chronic inflammatory diseases.

## Introduction

Despite significant advances in the prevention and management of atherosclerotic CVD, they are expected to remain as the major cause of human mortality and disability worldwide (1–3). Whereas the public health situation and economic impact of CVD claims for novel therapies, vascular drug development has paradoxically declined in the last two decades owing to the intrinsic complexities of CVD, as compared to other life-threatening and costly chronic diseases like cancer (3). Although recent advances in the understanding of atherosclerosis highlight the complexity of the pathophysiological events underlying the development of atheromatous plaques and subsequent vascular complications, novel opportunities for therapeutic interventions have also been revealed (see editorial (2) and compendium articles therein). An overwhelming amount of information, mostly derived from animal studies, has implicated the immune system and the inflammatory response as key drivers/regulators of atherosclerotic CVD. On the one hand, macrophages and T cells play a critical role in atherosclerosis progression by triggering and maintaining proatherogenic innate and adaptive immune responses, involving proinflammatory mediators such as IL-1, IL-6, or IFNγ. On the other hand, concomitant atheroprotective versions of the innate and adaptive responses, such as healing-promoting macrophages and regulatory T (Treg) cells, develop to counterbalance chronic inflammation and promote its resolution via regulatory mediators such as IL-10 (4–6). The translational relevance of this immune hypothesis of atherogenesis remained controverted during decades, since evidence in humans was mostly associative and circumstantial, rather than causal (7). However, the CANTOS study has ended this controversy. It has demonstrated that neutralization of IL-1β, a key proatherogenic/proinflammatory cytokine, significantly reduced the inflammatory burden and protected from secondary CVD events (8), providing a “proof of principle” for targeting of immune pathways, via anti-inflammatory and immunomodulatory agents, as a therapeutic intervention against CVD in humans (9, 10).

Whilst direct blocking of proinflammatory atherogenic pathways has been the obvious focus in the search for a CVD immunotherapy (8), a less explored alternative form of immunomodulation is the reinforcement/restoration of the natural atheroprotective mechanisms that become insufficient as atherosclerosis progresses. Among the immune cell types that promote atheroprotection, Tregs have emerged as dominant players (4–6, 11). Tregs not only exert classical regulatory functions on effector proatherogenic immune cells, but also influence cholesterol metabolism, plaque stability, foam cell formation and endothelial response, participate in plaque regression and tissue repair after ischemic events, and contribute to the athero/cardioprotective effects of statins, vaccination and inflammation resolution inducers (4–6, 12, 13). Thus, from a therapeutic perspective, it is difficult to envision a cell type, other than Treg, that could impact atherogenic responses in a more essential manner. Although a variety of methods have been proposed to restore Treg numbers and functions, including cell therapy, biologicals or particle-based sophisticated approaches (11, 12, 14, 15), their implementation for routine clinical practice is hampered by high costs and/or complexity, making them poorly realistic for widespread clinical use. Small molecules represent an alternative that might offer interesting advantages such as lower cost, which could facilitate preclinical and clinical development, and most importantly, accelerate implementation to clinical settings and its eventual use by the majority of patients. Investigations that explore small molecule route/dose/scheme of administration, delivery strategies and novel combinatory formulations could be of particular interest.

Many synthetic or naturally occurring small molecules have been reported as Treg inducers *in vitro* and *in vivo*, including immunosuppressive/anti-inflammatory drugs (such as Dexamethasone, Dexa, Rapamycin and Acetylsalicylic Acid), antioxidants (such as the natural polyphenols Curcumin and Quercetin, or the synthetic *N*-Acetylcysteine) and vitamins (such as Vitamin D, VitD, Vitamin A, Vitamin C, and Vitamin E) (16–21). Interestingly, but not surprisingly, the Treg-inducing capacity of these compounds is largely explained by their ability to condition antigen presenting cells (APC) toward a tolerogenic form. Many reports have shown that dendritic cells (DC) incubated *in vitro* with those compounds were potent Treg inducers *in vitro*, or *in vivo* upon adoptive transfer, demonstrating that DC-intrinsic effects are sufficient to explain their tolerogenic capacity. Based on this, we reasoned that a low-dose local (non-systemic) delivery of these compounds could be sufficient to condition local or draining lymph node (dLN) DC to acquire tolerogenic functions and Treg-inducing capacity *in vivo*. A low-dose but repetitive subcutaneous scheme would assure a sustained local/regional activity with little systemic distribution, which appeared particularly appropriate and convenient for agents potentially toxic and/or with a very restricted biodisponibility given their physical-chemical properties. A pioneering work by Kang and collaborators (21) demonstrated that subcutaneous (sc) delivery of low-dose Dexa, in combination with an antigen, efficiently induced tolerogenic DC (tolDC) and antigen-specific Tregs that restored tolerance and impacted disease, providing further support to our rationale. Thus, we initiated the search for a tolDC/Treg-inducing small molecules that exerts atheroprotection under the administration conditions described above in dyslipidemic mice, and found that combined VitD/Dexa is a promising formulation.

## Materials and Methods

For detailed information, see Supplementary Material.

### Mice, Treatment and Samples

**FIGURE 1:**
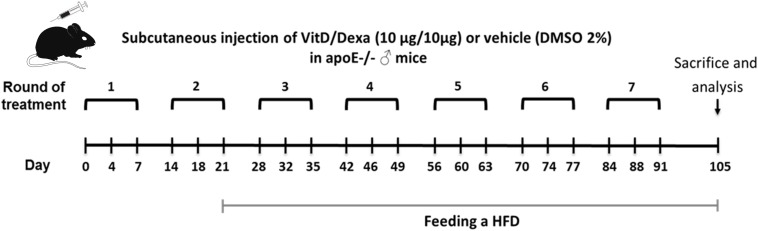
Schematic illustration of the experimental setup. Apolipoprotein E-deficient (ApoE^–/–^) mice were treated as indicated and fed a high-fat diet (HFD) during 12 weeks, as shown. Animals were subsequently sacrificed to remove the spleen (SP), popliteal (PLN), mediastinal (MLN) lymph nodes, and hearts-aortas for further analysis. Blood was also taken to obtain serum samples.

Male ApoE^–/–^ mice were injected sc into the footpad with the combination of VitD and Dexa (10 μg each), or vehicle, and fed a chow or a HFD, as shown in Figure 1. This scheme was designed in order to maximize the chances of evidencing a protective effect in the case it existed. After sacrifice (euthanasia with Ketamine/Xylazine 100/10 mg/kg, ip), spleens (SP), popliteal LN (PLN), blood and hearts-aortas were collected. Mediastinal LN (MLN) were also collected in some experiments. Wild-type C57BL/6 and BALB/c mice were also used in some experiments (see Supplementary Material for details) and euthanized as above. The institutional ethical committee (Comité Institucional para el Uso y Cuidado de los Animales de Experimentación, CICUAL) approved all the experiments, and procedures were performed according to the guidelines from Directive 2010/63/EU of the European Parliament.

### Immunological Analysis

SP and LN cell suspensions were obtained to analyze the frequency of tolerogenic (IL-10-producing) APCs, Tregs (IL-10-producing CD4^+^ T cells, called here Tr1-like Tregs and forkhead box P3^+^ (FOXP3^+^) CD4^+^ T cells, called here FOXP3^+^ Tregs), and IFNγ-producing CD4^+^ T cells (called here Th1 cells) by flow cytometry. SP cell suspensions were used to assess the amount of IFNγ and IL-10 secreted after stimulation with Concanavalin A (ConA). See Supplementary Material for details.

### Atheroprotection and Serum Analysis

Cryosections from the aortic root were used to quantify atherosclerotic lesion size, lipid deposition area, and macrophage and T cell infiltration by H/E, Oil red O staining, and immunohistochemistry. Serum samples were obtained to quantify the circulating levels of the lipid peroxidation product malondialdehyde (MDA), cholesterol, triglycerides, calcium, and glucose (22).

### Immunosuppressive Effect of VitD/Dexa *In vivo*

C57BL/6 mice were also used to evaluate the capacity of VitD/Dexa treatment to reactivate a latent infection with *Leishmania* parasites, by following the size of the cutaneous lesions and the amounts of viable parasites at the site of infection. Further details can be found in Supplementary Material.

### Statistical Analysis

Data were tabulated and analyzed using GraphPad Prism 6.0 software. Normality and equality of variances were tested by D’Agostino-Pearson test and Levene test, respectively. Student’s *t*-test or Mann-Whitney *U*-test were used for comparison between unmatched two groups. Two-way ANOVA followed by Bonferroni’s post-test was conducted in multiple group comparisons. *P* < 0.05 was considered significant.

## Results

### Combined VitD/Dexa as a Promising Treg-Inducing Formulation *In vivo*

We began by performing a pilot *in vivo* screening in which we tested a collection of nine small molecules with documented Treg-inducing capacity (16–21) using a low-dose repetitive sc injections. In a first experiment we confirmed that all compounds tested were indeed Treg inducers after two rounds of treatment (Supplementary Figure S1A). In a second experiment, in which the number of rounds was increased to three, we found that the combination of VitD and Dexa induced the highest percentage of FOXP3^+^ CD4^+^ T cells when injected either in absence or presence of the atherosclerosis-relevant antigen LDL (Supplementary Figure S1B).

### In Hypercholesterolemic ApoE^–/–^ Mice, Low-Dose sc VitD/Dexa Promotes IL-10 Production by DC and Other APC

**FIGURE 2:**
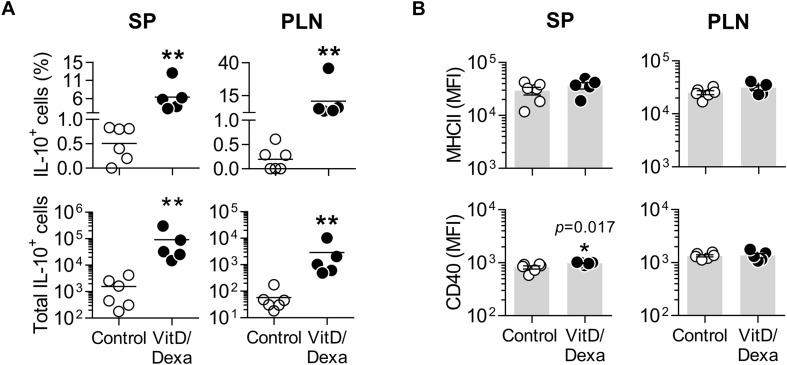
VitD/Dexa promotes IL-10 production by cDC in ApoE^–/–^ mice. SP and PLN cell suspensions were stimulated with PMA/ionomycin and stained with the appropriate mAbs to quantify the intracellular IL-10 accumulation and the surface expression of major histocompatibility complex class II (MHC-II) and CD40 in cDC (CD11c^*hi*^ MHC-II^+^ cells) by flow cytometry, as explained in Supplementary Figure S2A. The percentages (upper panel) and absolute numbers per organ (lower panel) of IL-10-producing cDC are shown **(A)**. The surface density of the maturation markers MHC class II (upper panel) and CD40 (lower panel) was also analyzed and reported as the mean of fluorescent intensity, MFI **(B)**. Lines/bars represent the mean **(A)** or the mean ± SEM **(B)** from 5 to 6 mice/group. Representative from two experiments. *P < 0.05, **P < 0.01 (Mann-Whitney test). Note that although MFI bars of VitD/Dexa and control groups in CD40 SP [**(B)**, lower panel, left] appear visually similar as a result of the log scale, statistic comparison revealed significance.

The result of our screening was interesting, since cross-talk between the glucocorticoid receptor (GR) and VitD receptor (VDR), and the synergistic interaction between their ligands have been described (23–25), suggesting that novel combined re-formulations of these agents might offer novel possibilities for the optimization of their immunomodulatory capacities, with better efficacy and safety. In fact, the power of combined VitD/Dexa to induce tolDC/Treg *in vitro* has been well documented (26, 27); however, surprisingly, the prophylactic/therapeutic potential of combined VitD/Dexa has been poorly explored *in vivo*. Based on this, we continued with a more detailed *in vivo* characterization of the immunoregulatory and atheroprotective capacity of VitD/Dexa in dyslipidemic mice. ApoE^–/–^ mice were treated with VitD/Dexa (10 μg each) or vehicle and fed a HFD as depicted in Figure 1, and the frequency of tolDC in lymphoid organs was determined by flow cytometry at the end of the experiment. Since IL-10 production is a key phenotypic and functional marker of the tolDC induced by VitD/Dexa (26, 27), we evaluated the production of this cytokine by CD11c^*hi*^ MHC-II^+^ cells (conventional DC, cDC) regionally in the PLN, the LN draining the site of drug administration, as well as systemically (in the SP) with a gating strategy that excluded other cell types (including B cells, Supplementary Figure S2A). Our results indicated that VitD/Dexa treatment significantly increases the percentage and absolute number of regional and systemic IL-10-producing cDC (Figure 2A). Because major histocompatibility complex class II (MHC-II) and costimulatory molecules are also important APC functional markers and it is known that VitD and Dexa alter their surface abundance (26, 27), we continued with our *ex vivo* analysis by determining the expression of MHC-II and CD40 on cDC. The overall density of these maturation markers in cDCs from VitD/Dexa-treated animals was slightly higher than in control mice, with statistical significance confirmed only for CD40 in the spleen (Figure 2B). Thus, in dyslipidemic mice, VitD/Dexa treatment does not appear to substantially alter surface MHC-II and CD40 expression, but it improves the capacity of cDC to produce the regulatory mediator IL-10.

**FIGURE 3:**
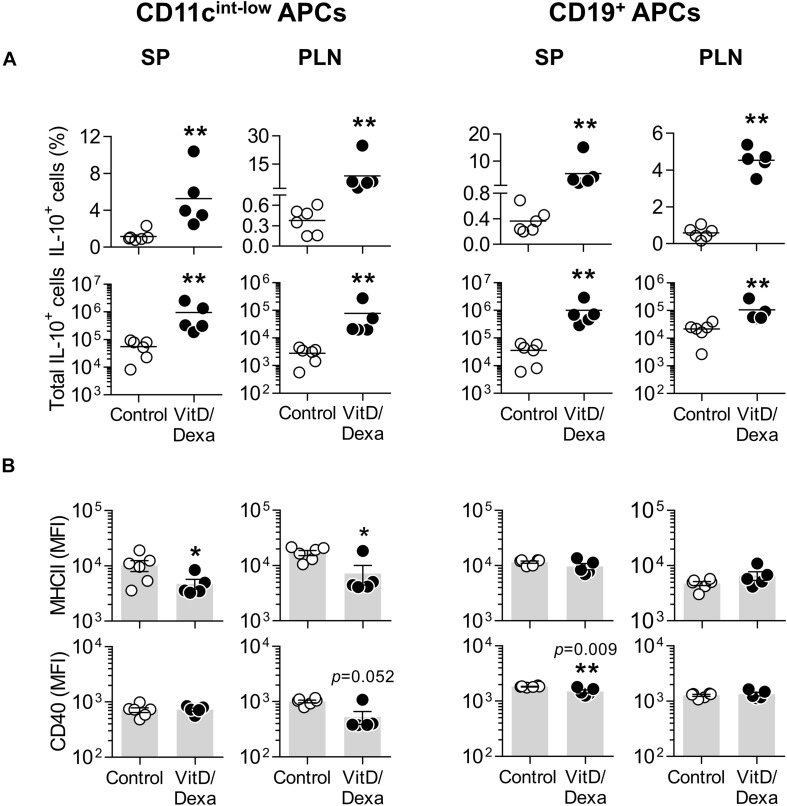
Other APC also upregulate IL-10 production in response to VitD/Dexa *in vivo*. SP and PLN cell suspensions were stimulated with PMA/ionomycin and stained with the appropriate mAbs to quantify the intracellular IL-10 accumulation and the surface expression of MHC class II and CD40 in CD11c^*int*−^^*low*^ MHC-II^+^ non-B APCs (left panel) and CD19^+^ B cells (right panel) by flow cytometry, as explained in Supplementary Figures S2A,B. The percentages (upper panel) and absolute numbers per organ (lower panel) of IL-10-producing cells are shown **(A)**. The surface density of the maturation markers MHC class II (upper panel) and CD40 (lower panel) was also analyzed and reported as the MFI **(B)**. Lines/bars represent the mean **(A)** or the mean ± SEM **(B)** from 5 to 6 mice per group. Representative from two experiments. *P < 0.05, **P < 0.01 (Mann-Whitney test). Note that although MFI bars of VitD/Dexa and control groups in CD40 SP B cells [**(B)**, CD19^+^ APC column, lower panel, left] appear visually similar as a result of the log scale, statistic comparison revealed significance.

Fluorescence-activated cell sorting analysis also allowed us to assess the production of IL-10 in other non-cDC APC, including B cells (by using the CD19 marker; Supplementary Figure S2B) and the non-B non-CD11c^*hi*^ MHC-II^+^ cells, which comprises mainly monocyte/macrophages and less abundant DC subpopulations (such as plasmacytoid DC and monocyte-derived DC; Supplementary Figure S2A). Interestingly, these two groups of APCs also expressed more IL-10 in the PLN and the spleen when mice were treated *in vivo* with VitD/Dexa (Figure 3A). Regarding the maturation marker expression, VitD/Dexa induced an overall reduction in the surface density of MHC-II and CD40 on the non-B CD11c^*int*^^−*low*^ MHC-II^+^ APCs (Figure 3B, left panel) and a weaker inhibitory effect only on CD40 expression by splenic CD19^+^ B cells (Figure 3B, right panel). Altogether, findings presented so far indicate that although exposure to low-dose VitD/Dexa might have distinct effects on the maturation/activation status of different APC *in vivo*, all APC subsets become more efficient producers of the regulatory cytokine IL-10.

### VitD/Dexa Treatment Promotes Treg Cells *In vivo*

**FIGURE 4:**
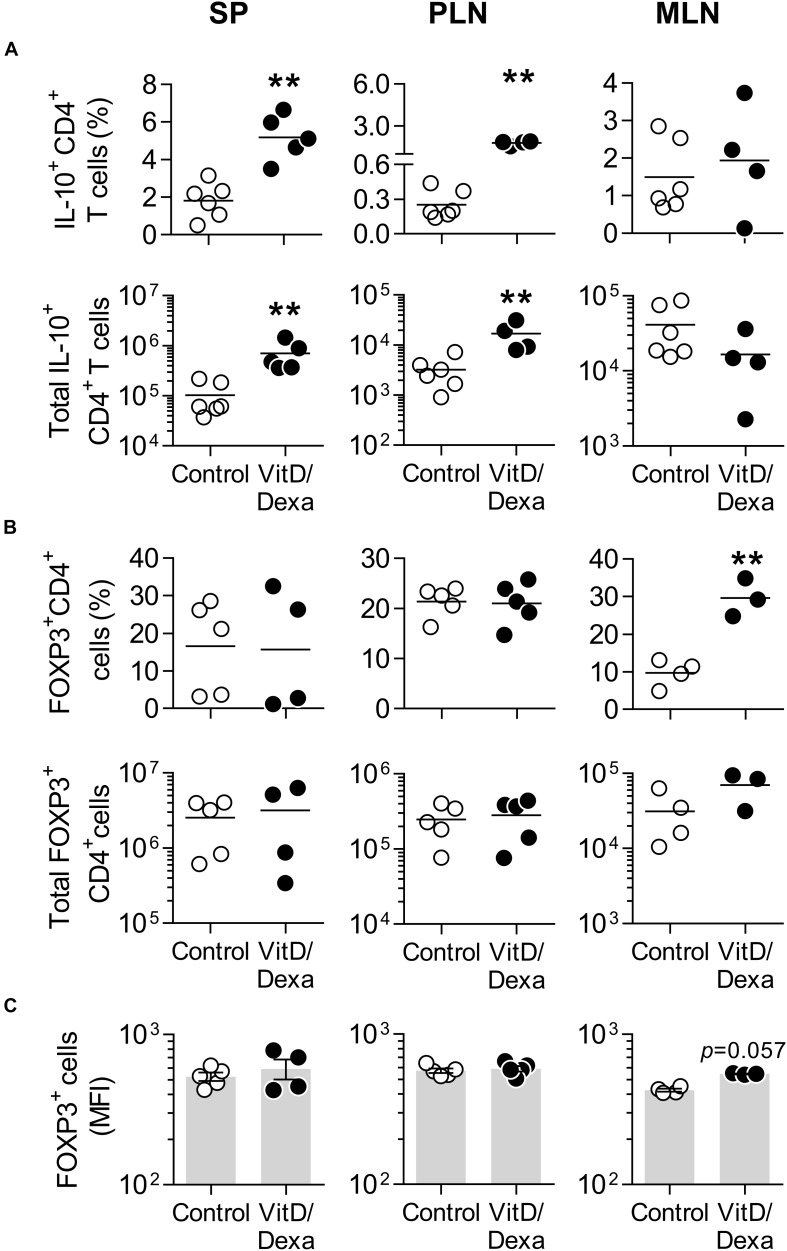
VitD/Dexa promotes IL-10-producing CD4^+^ T cells *in vivo*. The amounts of IL-10^+^ CD4^+^ T cells **(A)** and forkhead box P3^+^ (FOXP3^+^) CD4^+^ Treg cells **(B)** were determined by flow cytometry in PMA/ionomycin-stimulated SP, PLN and MLN cell suspensions from VitD/Dexa- and vehicle-treated ApoE^–/–^ mice (see gating strategies in Supplementary Figures S3A,B). The frequency (upper panel) and total numbers per organ (lower panel) are shown. The cellular abundance of FOXP3 was also determined on the FOXP3^+^ CD4^+^ cells and graphed as the MFI **(C)**. Lines/bars represent the mean **(A,B)** or the mean ± SEM **(C)** from 3 to 6 mice/group. Results are representative of two **(A)** or three **(B,C)** independent experiments. **P < 0.01 (Mann-Whitney test).

Vitamin D3 and/or Dexa-conditioned DC are potent inducers of the two major Treg subsets, FOXP3^+^ and Tr1-like Tregs, both *in vitro* and *in vivo* (26, 27). This, together with our observation that combined VitD/Dexa induces IL-10-producing DC/APC *in vivo* (Figures 2, 3), prompted us to analyze the amounts of IL-10-producing CD4^+^ T and FOXP3^+^ CD4^+^ T cells *ex vivo* (Supplementary Figure S3). Results confirmed that VitD/Dexa treatment markedly increased the relative and absolute number of IL-10-producing CD4^+^ T cells (Tr1-like Tregs) in the SP and PLN (Figure 4A). On the other hand, no changes in the FOXP3^+^ CD4^+^ T cells (FOXP3^+^ Treg) subset were observed in these lymphoid organs (Figures 4B,C). We extended our Treg analysis to MLN, the secondary lymphoid organs draining the heart, and found that animals treated with VitD/Dexa had increased percentages of FOXP3^+^ but not of Tr1-like Tregs when compared to control animals (Figures 4A,B). The analysis of the abundance of the FOXP3 marker (as assessed by the mean of fluorescent intensity, MFI) in CD4^+^ T cells from MLN indicated a non-statistically significant trend to higher level in VitD/Dexa-treated mice compared to control mice (Figure 4C). In summary, this set of analysis confirmed that VitD/Dexa also induces Tr1-like Tregs in dyslipidemic mice.

### VitD/Dexa-Treated Mice Have Less IFNγ^+^-Producing T Cells

**FIGURE 5:**
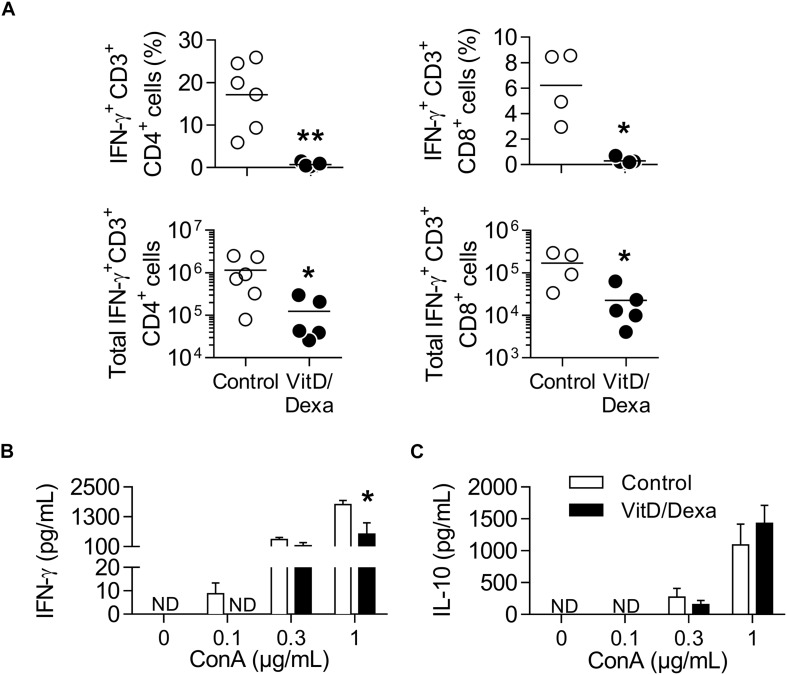
VitD/Dexa-treated mice have less IFNγ-producing CD4^+^ and CD8^+^ T cells. PMA/ionomycin-stimulated splenocytes were stained with the appropriate mAbs to quantify the frequency (upper panel) and total numbers per SP (lower panel) of IFNγ-producing CD3^+^ CD4^+^ (left panel) and CD3^+^ CD8^+^ (right panel) T cells **(A)** as explained in Supplementary Figures S4A,B. Total splenocytes were also cultivated in the presence of ConA at the indicated concentration during 48 h and the amount of IFNγ **(B)** and IL-10 **(C)** released to the supernatant was determined by ELISA. Results in **(A)** are of individual mice and lines represent the mean from 4 to 6 mice/group. Data in **(B)** are shown as the mean ± SEM from 4 to 5 mice/group. ND, not detected. *P < 0.05, **P < 0.01 [**(A)** Mann-Whitney test; **(B)** Two-way ANOVA test followed by Bonferroni’s post-test].

**FIGURE 6:**
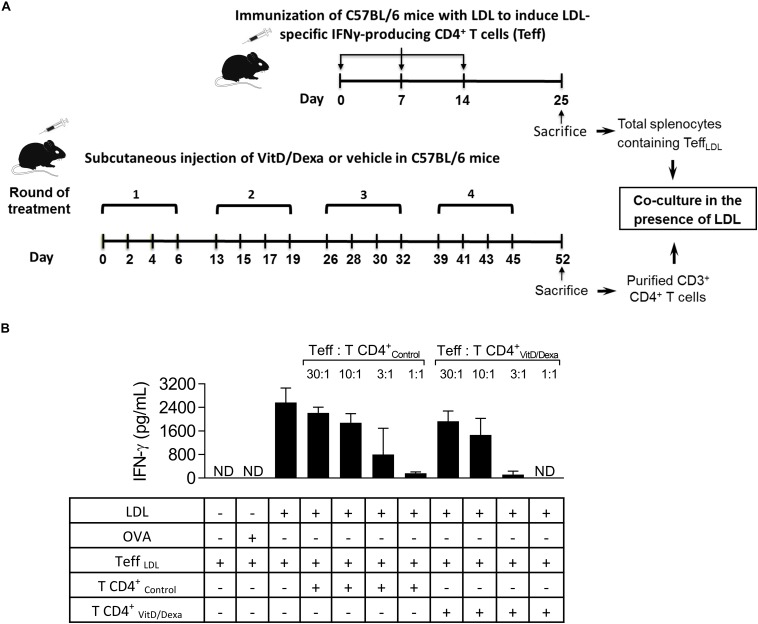
VitD/Dexa promotes CD4^+^ T cells that are suppressive *ex vivo.* Wild type C57BL/6 mice were treated with VitD/Dexa or vehicle, and CD3^+^ CD4^+^ T cells were purified by FACS-sorting to be co-cultured with total SP cells from LDL-vaccinated C57BL/6 mice (containing IFNγ-producing effector T cells; Teff), in the presence of the relevant antigen for 72 h **(A)**. The suppressive activity of the CD4^+^ T cells was determined by the amount of IFNγ released to the medium at different Teff:TCD4^+^ ratios **(B)**. CD4^+^ T and Teff were obtained from pooled splenocytes from three mice. OVA was also included as a control of non-relevant antigen stimulation. Data are shown as mean ± SD from pooled triplicate cultures. ND, not detected. Note that whereas in 1:1 Teff:TCD4^+^-Control co-cultures the mean of IFNγ expression was 168 pg/mL, in 1:1 Teff:TCD4^+^-VitD/Dexa co-cultures IFNγ was not detectable (which means less than 3.1 pg/mL).

IFNγ^+^-producing T cells are major drivers of local and systemic inflammation and have a pivotal role in atherosclerosis development and in CVD (4–6). Because Tregs inhibit the proatherogenic Th1 response (4–6, 11, 14), we next investigated whether VitD/Dexa treatment impacted the numbers of pro-inflammatory T cells in the spleen (following the gating strategy shown in Supplementary Figure S4). ApoE^–/–^ mice exposed to the VitD/Dexa formulation had less IFNγ-producing CD4^+^ (Th1) cells compared to vehicle treated mice (Figure 5A, left panel). VitD/Dexa treatment also reduced the percentage of IFNγ^+^ CD8^+^ T cells (Figure 5A, right panel), which have been recently suggested as promoters of atherosclerosis by controlling monopoiesis and circulating monocytes (28). Moreover, when splenocytes from VitD/Dexa-treated mice were stimulated with the polyclonal T cell activator ConA, IFNγ secretion was lower than that observed in splenocytes from vehicle-treated mice (Figure 5B). Conversely, when IL-10 production was quantified in the same supernatants, a non-significant increasing trend was observed in VitD/Dexa-treated mice at the highest ConA concentration (Figure 5C). These results, therefore, confirm that sc low-dose VitD/Dexa also blunts IFNγ response in dyslipidemic mice. Thus far, our observations are compatible with a scenario in which VitD/Dexa treatment promotes a Treg-enriched microenvironment in secondary lymphoid organs such as SP and PLN, which in turn mediates the suppression of the Th1 response. We tested this idea by assessing the suppressive capacity of the CD4^+^ T cells induced *in vivo* by VitD/Dexa treatment. Supernatants of total splenocytes from LDL-vaccinated C57BL/6 mice (containing IFNγ-producing Teff cells) co-cultured in the presence of LDL with purified CD4^+^ T cells from VitD/Dexa-treated mice had less IFNγ than supernatants of Teff cells co-cultured with CD4^+^ T cells from vehicle-treated mice, indicating a greater suppressive activity on the cellular LDL-specific immune response (Figures 6A,B). Together, these results support the hypothesis that the VitD/Dexa formulation promotes suppressive Tregs that might be able to reduce atherosclerosis-related inflammation.

### VitD/Dexa Protects Against Atherosclerotic Lesion Development in Dyslipidemic Mice

**FIGURE 7:**
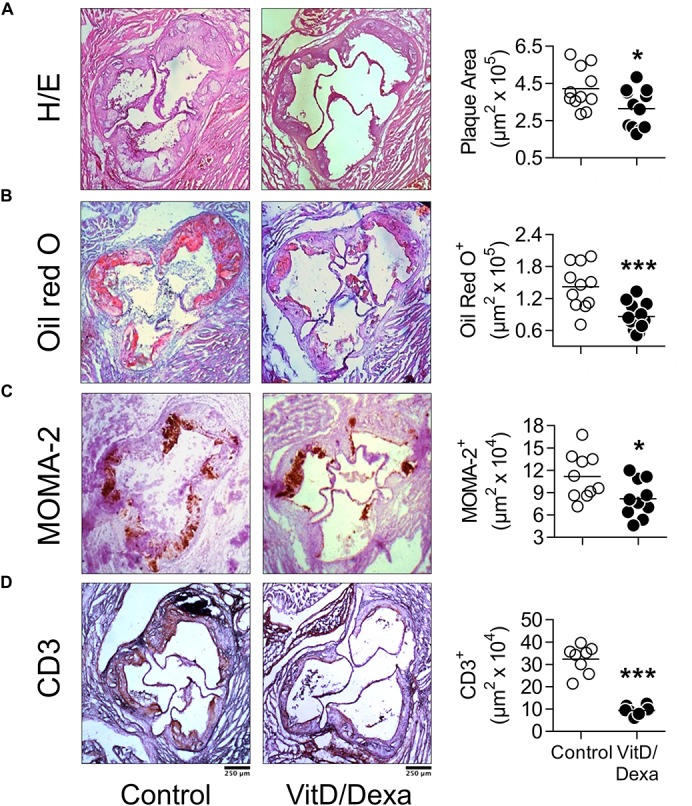
VitD/Dexa attenuates the development of atherosclerosis in dyslipidemic mice. ApoE^–/–^ mice were treated with VitD/Dexa (10 μg) or vehicle and fed a HFD, as shown in Figure 1. Cryosections from the aortic root were stained with H/E **(A)** and Oil red O **(B)** or probed with macrophage (CD68)- and T cell (CD3)-specific antibodies for immunostaining **(C,D)**. Representative micrographs for each treatment group are shown (left panel). The areas of the atheromatous lesions, lipid deposition as well as the macrophage and T cell infiltrate were calculated with image analysis software. The black bar on the left panel represents 250 μm. Each point represents the average area per mouse (7–9 sections/mouse), and bars represent the mean (right panel). Representative results of three independent experiments are shown. *P < 0.05; ***P < 0.001 (Student’s *t*-test).

Having demonstrated that VitD/Dexa increases regulatory and reduces proinflammatory immune cell response in ApoE^–/–^ mice, our next step was to assess the development of the atherosclerotic lesions. Remarkably, the analysis of aortic root sections revealed a significant atheroprotective effect of VitD/Dexa-treatment, as evidenced by the reduction in the size of the atheromatous lesions (Figure 7A). These mice also had less lipid deposition in the aortic roots (Figure 7B), which resulted intriguing, since no lipid-lowering effect could be demonstrated by VitD/Dexa-treatment (Table 1). Moreover, no antioxidant effect could be demonstrated, as the circulating levels of the lipid peroxidation product MDA were comparable between VitD/Dexa- and vehicle-treated mice (Table 1). These results, together with the significant reduction in the amount of macrophages and T cells infiltrating atherosclerotic lesions (Figures 7C,D), and the previously presented evidence of regulatory immune cells induction and Th1 inhibition (Figures 2–5), suggest that atheroprotection by sc low-dose VitD/Dexa operates via immune modulation rather than lipid-modifying or antioxidant systemic effects.

**TABLE 1:** Body weight, serum analyses and lymphoid organ cell counts in dyslipidemic ApoE^–/–^ mice.

Parameters	Control	VitD/Dexa	*P*-value	*P*-value summary
**Body weight, g (across the treatment)**				
Week 1	25.38 ± 3.46	23.63 ± 3.05	0.20	ns
Week 4	27.88 ± 2.25	26.43 ± 1.91	0.10	ns
Week 8	30.41 ± 3.42	30.82 ± 3.31	0.77	ns
Week 12	35.38 ± 4.60	34.26 ± 6.19	0.62	ns
Week 16	36.70 ± 4.935	33.20 ± 4.50	0.08	ns
**Serum analysis**				
Cholesterol Total, mg/dL	1301.00 ± 423.10	1427.00 ± 384.30	0.452	ns
Triglyceride, mg/dL	142.30 ± 88.33	130.10 ± 23.03	0.636	ns
Malondialdehyde (MDA), nmol/mg protein	2.75 ± 1.06	3.93 ± 2.04	0.117	ns
Glucose, mg/dL	398.10 ± 89.62	414.70 ± 105.80	0.691	ns
Calcium, mg/dL	8.77 ± 0.36	9.45 ± 0.95	0.037	*
**Lymphoid organ cell counts**				
**Viability of cell suspensions, (%)**				
SP	88.35 ± 8.09	93.91 ± 1.51	0.058	ns
PLN	92.05 ± 1.68	94.52 ± 3.06	0.032	*
MLN	85.13 ± 6.45	85.65 ± 8.35	0.883	ns
**Number of total leukocytes/organ**				
SP	1.43*E* + 08 ± 4.55*E* + 07	1.59*E* + 08 ± 6.50*E* + 07	0.900	ns
PLN	6.80*E* + 06 ± 3.09*E* + 06	4.67*E* + 06 ± 2.13*E* + 06	0.097	ns
MLN	1.93*E* + 06 ± 8.48*E* + 05	1.30 + 06 ± 1.02*E* + 06	0.173	ns
**Number of CD4^+^ T cells/organ**				
SP	1.35*E* + 07 ± 1.15*E* + 07	2.62*E* + 07 ± 2.20*E* + 07	0.114	ns
PLN	1.40*E* + 06 ± 7.20*E* + 05	1.70*E* + 06 ± 1.39*E* + 06	0.542	ns
MLN	1.85*E* + 05 ± 7.73*E* + 04	2.83*E* + 05 ± 1.41*E* + 05	0.079	ns

Non-fasted serum samples were collected from animals. Data are presented as the mean ± SD (representative of two independent experiments, 11 to 12 mice per group, Student’s *t*-test, *P < 0.05).

### Repetitive Low-Dose VitD/Dexa Treatment Does Not Induce Overall Toxicity or Immunosuppression

**FIGURE 8:**
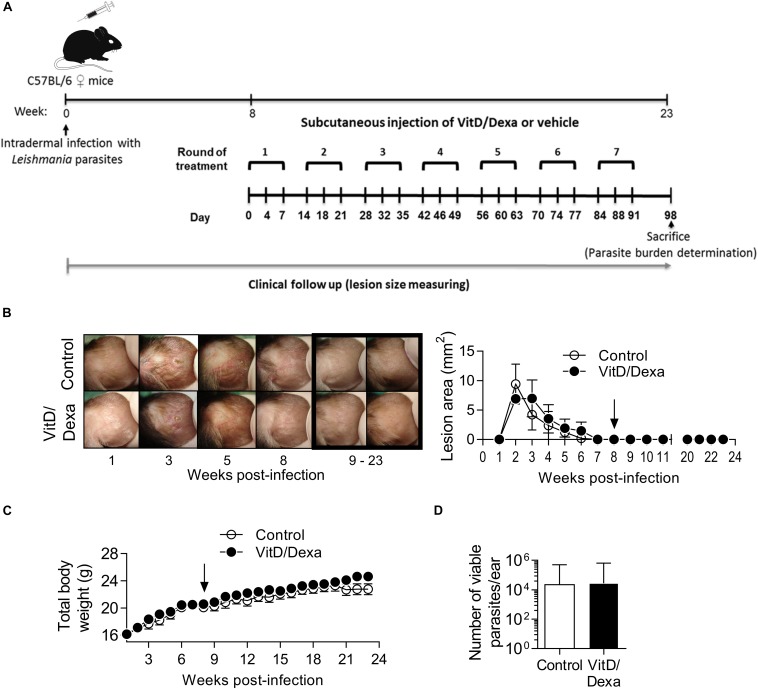
No evidence of immunosuppression in mice treated with an atheroprotective dose/scheme of VitD/Dexa. Wild-type C57BL/6 mice were infected with 1 × 10^5^ stationary-phase *Leishmania panamensis* promastigotes. Once mice were completely cured and entered the latent phase of infection (8 weeks after infection) an atheroprotective treatment with of VitD/Dexa was administered **(A)**. Representative photographs from infected ears in the experimental groups are shown [**(B)**, left]. Post-treatment representative photographs are marked. A clinical follow-up was performed by measuring the lesion size of infected ears weekly [**(B)**, right]. Mice were also weighted weekly **(C)**. At the end of the experiment (23 weeks) the parasite burden in the infected ears was determined **(D)**. The arrows indicate the initiation of the treatment. Results are presented as the mean ± SEM **(B,C)** or the geometric mean ± 95%CI **(D)** from 5 mice/group. Representative of two experiments. No statistically significant differences were found between the two groups [**(B,C)** Two-way RM ANOVA; **(D)** Mann-Whitney test].

Depending on the dose and extension of the treatment, both VitD and Dexa are potentially deleterious and might induce adverse effects such as disturbances in calcium, glucose or lipid metabolism, or immunosuppression. It was noteworthy that ApoE^–/–^ mice treated with VitD/Dexa gained weight similarly to vehicle-treated mice (Table 1) and had healthy appearance and normal behavior, just like control mice. Furthermore, at the end of the experiment, no changes on serum glucose and lipid levels, or major effects on lymphoid cell viability or total CD4^+^ T cell numbers were observed in VitD/Dexa treated mice (Table 1). Although a mild (7%) but significant increase in serum calcium levels was observed in VitD/Dexa-treated mice (Table 1), these levels (9.45 ± 0.95 mg/dl) were comparable to reported reference values in 16-week-old ApoE^–/–^ mice (2.28 ± 0.22 mM or 9.12 ± 0.88 mg/dl) (29), as well as to the calcium serum levels reported by Charles River Laboratories in C57BL/6 mice (IC 95% 9.7–12.5 mg/dl). In addition, no clinical signs of hypercalcemia (e.g., dehydration or weight loss, as shown above) were detected. A major safety concern of pharmacological anti-inflammatory agents such as VitD and specially Dexa is their potential immunosuppressive effect, which might impair effector immune mechanisms against tumors and infections. To gain insights into the potential immunosuppressive effects of low-dose VitD/Dexa, we examined its impact on cutaneous leishmaniasis, a chronic and persistent infection in which immunosuppression promotes disease reactivation. In a murine model of cutaneous leishmaniasis by *Leishmania panamensis* (*Lp*) established in our laboratory, we have observed that BALB/c mice challenged with infective parasites develop progressive ulcerative cutaneous lesions whereas C57BL/6 mice develop mild self-limited lesions. Although cured C57BL/6 mice become immune to reinfections, they harbor persistent parasites at the site of infection, rendering them suitable models for assessing the reactivation of a latent infection. C57BL/6 mice (and BALB/c mice, as a control for the inoculum infectivity) were inoculated intradermally in the ears to subsequently monitor lesion development (Figure 8A). As expected, BALB/c mice developed a severe disease (Supplementary Figures S5A,B) while C57BL/6 mice showed a mild self-limited clinical form that cured within 6–8 weeks postinfection (Figures 8B). Once C57BL/6 mice completely healed the lesions, they were treated with VitD/Dexa using the same route/dose/scheme that resulted in atheroprotection in ApoE^–/–^ mice (Figures 1, 8A) and monitored weekly to detect any sign of lesion reactivation. Similarly to vehicle-treated mice, the ears of VitD/Dexa-treated animals presented a normal, healed and healthy appearance, with no redness, swelling, or other clinical signs of disease reactivation during the whole experimentation period (Figure 8B). Both groups of mice also gained weight in a similar manner (Figure 8C), had a healthy overall appearance and normal behavior, and did not exhibit piloerection, lethargy, or signs of dehydration. Most importantly, and consistently with clinical observations, the number of viable parasites in the ears of VitD/Dexa-treated mice did not differ from control mice (Figure 8D), confirming the absence of parasite reactivation. Thus, these results indicate that the effector immune mechanisms that maintained lesion-free parasite latency remained unaffected after chronic low-dose VitD/Dexa treatment.

## Discussion

Although significant efforts have been made in the last years to develop useful immunomotherapeutic agents for chronic inflammatory conditions, such as CVD, two major problems remain (30). First, safety issues such as immunosuppression that might increase the susceptibility to infections and tumors (8, 31). And second, the high costs [for example of biological drugs; (32, 33)] and the logistic and technical limitations [for example of cellular therapies; (34)] that make unrealistic the translation of the agents to routine prophylactic or therapeutic clinical use. VitD and Dexa, listed by WHO as essential medicines, are two of the most prescribed drugs by clinicians, are widely available at an exceptionally meager cost, and importantly, there is plenty of scientific information on their pharmacology, toxicology, efficacy and safety. Thus, the repurposing and reformulation of these agents could be inexpensive and rapid, and their clinical use realistic. Here we report that our attempts to find a simple, safe and inexpensive alternative Treg-inducing small molecule led us to test and confirm that the local low-dose repetitive delivery of a tolDC/Treg-inducing formulation that combines VitD and Dexa promotes atheroprotection in ApoE^–/–^ mice.

Current therapeutic use of VitD and Dexa in the clinical practice (high dose, oral/muscular delivery) implies systemic distribution with the expected consequences, beneficial and toxic, dictated by the pharmacological response of the ubiquitously expressed VDR and GR (35–37). Given the physical-chemical properties (very low water solubility) we reasoned that local (e.g., footpad, sc route) low-dose (10 μg) treatment using a repetitive but spaced scheme (rounds every other week) would provide biologically relevant concentrations of VitD/Dexa at local/regional level rather than systemically, restricting their effects to the site of injection and dLN. Although a detailed pharmacological analysis is required to formally confirm the absence or the negligible circulation and distribution, all analysis performed (clinical, biochemical, redox, immunological and immunosuppressive effect; Table 1; Figure 8) indicated the lack of direct systemic effects of VitD/Dexa, including toxicity. In contrast, the desired immunological effects (induction of regulatory cells) on the dLN were evident (Figures 2–4). Moreover, in preliminary experiments, we have detected the induction of apoptotic cells in the dLN but not in the spleen after sc VitD/Dexa injection (data not shown), further supporting our hypothesis of regional VitD/Dexa delivery but negligible systemic exposure. These results confirmed that sc low-dose VitD/Dexa formulation is indeed a safe and non-immunosuppressive tolDC/Treg-inducer in mice.

Despite the hyperlipidemic and proinflammatory conditions characterizing ApoE^–/–^ mice, which are further accentuated by HFD feeding, the treatment with VitD/Dexa was able to efficiently induce IL-10 production in DCs and other APCs (Figure 2, 3), shifting CD4^+^ T cells response toward a less accentuated Th1 profile, and promoting an increased Treg response (Figures 4, 5) and atheroprotection (Figure 7). Our observations are in agreement with an increasing number of reports suggesting that interventions modifying the DC-T cell crosstalk under hyperlipidemic conditions, from the prevailing proimmunogenic form toward a more tolerogenic form, results in atheroprotection (4–6, 9–14, 30). Previous reports have indicated that low-dose sc Dexa promoted tolDC, increased Treg/Teff levels and protected mice from autoimmunity (21) and atherosclerosis (38) when coinjected with a disease related-relevant antigen. Also, that oral delivery of VitD promoted tolDC, Treg, and atheroprotection (39). Our work extends those findings by showing that a combined low-dose formulation of VitD/Dexa, delivered locally in the absence of coinjected antigen, delays atherosclerosis development. Although we did not directly compare the two single versus combined VitD/Dexa within the same trial, two pilot independent experiments revealed the dependency of the co-formulation for inducing significant atheroprotection (Supplementary Figure S6). This result, together with the improved Treg-inducing capability observed for the combined formulation in wild-type mice (Supplementary Figure S1) suggest a potential additive or synergistic atheroprotective effect for these two small molecules that requires further investigation. Considering the well demonstrated synergy between VitD and Dexa to induce tolDC and Treg *in vitro* (26, 27), it is to be evaluated whether atheroprotective effects of VitD/Dexa can be further optimized by refining the amounts and relation of the agents and the treatment scheme. Similarly, whether coinjection of a relevant atheroantigen such as LDL together with VitD/Dexa will lead to improved atheroprotection is unknown, but is currently being investigated in our laboratory.

Due to technical limitations, we were not able to investigate whether VitD/Dexa-induced Tregs were atheroantigen-specific. Nonetheless, CD4^+^ T cells from VitD/Dexa-treated mice were more effective suppressors of the LDL-stimulated Th1 effector response *ex vivo* compared to vehicle-treated mice (Figure 6), and no evidence of immunosuppression was observed after VitD/Dexa administration (Figure 8). Thus, we speculate that oxLDL/LDL present in plasma, lymph, and other fluids from wild-type mice, and more notably from HFD-fed ApoE^–/–^ mice, might have contributed to the tolerogenic conditioning of DCs at the draining site of VitD/Dexa injection, providing relevant atheroantigens, and possibly contributing to the subsequent induction of atheroantigen-specific Treg. Although this idea requires further experimental support, a similar phenomenon has been reported with the atheroprotective Treg-inducing adjuvant Alum (40). Thus, our results represent not only a preclinical proof of concept for a simple VitD/Dexa formulation as a potential form of immune intervention for atherosclerosis but also for a tolerogenic adjuvant platform that could be combined with any disease-relevant antigen to induce antigen-specific tolerance for prophylactic or therapeutic purposes.

The detailed mechanistic explanation of the immunomodulatory and atheroprotective effects described here requires further investigation to be fully established. A causal relation between IL-10 production by cDC and/or CD4^+^ T cells and atheroprotection will require appropriated tools, such as mice deficient in IL-10 production by hematopoietic cells or particular leukocyte types. Our results, however, are consistent with the role of IL-10 as a key antiatherogenic factor and with the atheroprotective effect of IL-10-conditioned DC (41) and Tr1-like Tregs *in vivo* (42). The question of whether other immune cell types contributed to atheroprotection also warrants further investigation. For instance, an eventual role of FOXP3^+^ Treg is intriguing since, opposite to Tr1-like Tregs, increased percentages were observed in MLN but not in PLN or spleens of dyslipidemic VitD/Dexa-treated ApoE^–/–^ mice (Figure 4B). This contrasted with the splenic FOXP3^+^ Treg response observed in non-dyslipidemic wild-type mice (Supplementary Figure S1B) and opens the possibility that Treg induction might be qualitatively different under dyslipidemic versus non-dyslipidemic conditions and that in VitD/Dexa-treated mice different Treg subsets might play distinct atheroprotective roles.

Similarly, the stimulation of IL-10 production by VitD/Dexa in non-cDC non-B APC (Figure 3A, left) is also interesting, since monocyte-derived DC appear to be important during atherosclerosis development and tolerogenic plasmacytoid DC are atheroprotective (43, 44). IL-10-producing tolerogenic macrophages have been identified in low-dose Dexa-treated mice (45) and, therefore, could also support the atheroprotection observed in our experiments. Also B cells are possible contributors to the atheroprotection reported here via IL-10 production (Figure 3A, right), according to the recent recognition of regulatory B cells (Breg) as potentially atheroprotective cells (46). We could not find differences in the serum levels of LDL-specific total IgG, or the subclasses IgG1 and IgG2c between treated and control mice (data not shown), suggesting that a B cell-dependent atheroprotective role could be more related to IL-10 production or, alternatively, to IgM natural antibodies. Our findings indicate that, in dyslipidemic mice, VitD/Dexa induces a regulatory network of IL-10-producing innate and adaptive immune cells that are likely mediators of atheroprotection. Moreover, preliminary evidence suggests that other leukocyte types (such as CD8^+^ T cells or NK cells) also upregulate IL-10 production (not shown), extending the repertoire of immune cells that produce IL-10 in response to the VitD/Dexa treatment *in vivo*. Accordingly, higher number of cells producing IL-10 in those mice is already evident in total splenocytes, without further gating of subpopulations (Supplementary Figures S7A,B). Taking this in consideration, the known role of IL-10 as inducer of MDSC, and the atheroprotective potential of MDSC (47), we also evaluated the abundancy of these cells. However, it was surprising that the numbers of MDSC did not substantially increase with VitD/Dexa treatment (Supplementary Figure S8). Thus, we hypothesize that the induction of an IL-10-producing leukocyte network is a key event underlying the atheroprotection reported here. The wide range of cells in which a regulatory program is triggered by this cytokine enabling the induction anti-inflammatory effects, such as infectious tolerance (48), would be sufficient to explain atheroprotection. More detailed future work will permit to analyze the kinetics and essentiality of each IL-10 producer for atheroprotection.

Our findings support a mode of action in which tolDC and Tregs are induced in the periphery (skin/dLN), and upon subsequent migration exert distant anti-inflammatory and atheroprotective effects. The presence of tolDC and Tregs in the spleen could be better explained by migration from the periphery rather than by splenic induction, as we don’t expect significant systemic exposure to VitD/Dexa. The spatiotemporal dynamics of VitD/Dexa-induced tolDC and Treg is also to be confirmed. However, according to current knowledge, a possible explanation is that VitD/Dexa-conditioning of DC might occur on skin migratory DC or on resident dLN DC. In both cases, tolDC will be present in dLN to mediate Treg induction. Also, Treg formation in the sc tissue cannot be ruled out, particularly in dyslipidemic mice where skin inflammation and abundant inflammatory lymphocyte infiltrate has been reported (49). Alternatively, dying cells induced locally by VitD/Dexa could be rapidly removed by efferocytic DC, triggering Treg induction. Both apoptosis induction and efferocytosis stimulation by these agents, as well as the connection between efferocytosis, Tregs, inflammation resolution and atheroprotection, have been documented (35, 36, 50, 51). As DC tolerogenic-conditioning via direct effects of the agents or indirectly via apoptosis/efferocytosis are not mutually exclusive, it is possible that a combination of them underlie the increased frequency of Treg observed in VitD/Dexa-treated mice.

How peripherally induced VitD/Dexa-conditioned tolDC and specially Tregs exert atheroprotection is also to be explored. The simplest explanation is that these regulatory cells migrate to the inflamed atheroma. However, our attempts to identify immune cells in the aortic roots by FACS were unsuccessful given the poor yields and limited viability of the cell suspensions obtained. When we analyzed the expression of some immune markers in whole abdominal aortas by qPCR, there was a trend showing reduced levels of expression of inflammatory markers such as IFNγ and MCP-1 in VitD/Dexa-treated mice (Supplementary Figure S9), which is concordant with the reduced inflammatory infiltrate observed in the aortic root (Figures 7C,D). However, mRNA levels of IL-10, and other Treg-related mediators such as TGF-β and FOXP3 exhibited the same tendency (Supplementary Figure S9). Although caution has to be taken with qPCR analysis from whole abdominal aortas, as results reflect the average abundance of mediators in both atheromatous and non-atheromatous areas of the artery, the confirmation of this finding would imply that tolDC and Treg induced by VitD/Dexa could have influenced more systemically in the spleen and relevant LN, rather than in the arteries. This is in line with the recent observation that whereas priming and amplification of effector IFNγ^+^ T cells occurs in the artery, Treg responses preferentially takes place in secondary lymphoid organs (52) and is concordant with our observations showing more Tregs in the spleen and MLN of VitD/Dexa-treated mice than in controls (Figure 4). Further research is needed to better characterize tolDC and Treg trafficking in VitD/Dexa-treated mice.

An important aspect that deserves further discussion is that the formulation utilized here was composed by cholecalciferol and Dexa, prepared in 2% DMSO. In contrast to Dexa, which directly triggers the GR, cholecalciferol is the VitD prohormone that requires the previous transformation into the active 1,25-hydroxy VitD hormone. VitD production, activation and inactivation have been extensively investigated in the context of its dominant function as a key regulator of mineral homeostasis. Sequential hydroxylation reactions at the positions 25 and 1 of cholecalciferol are known to occur in the liver and kidneys, respectively. In the context of the immunomodulatory actions, it is of great interest that immune cells not only express VDR, making them responsive to the active hormone, but also the key metabolizing enzymes, which enables them to transform cholecalciferol into active VitD (53). Particularly in the skin, where cholecalciferol is bioavailable via endogenous synthesis or diet, dendritic cells have been described as a local source of active VitD (54). Interestingly, under inflammatory conditions and/or through DC-T cell cross-talk, VitD-activating enzymes are upregulated, leading to increased local VitD activation (53, 54). Thus, the inflamed skin and dLN that characterizes dyslipidemic mice (49) are expected to be particularly capacitated to transform inactive VitD into the active form. Although this requires experimental confirmation by measuring inactive and active forms of VitD locally, and VitD-activating enzymes in immune cells, our results of tolDC and Treg induction (Figures 2, 4) and atheroprotection (Figure 7) are consistent with the idea of local activation of cholecalciferol upon sc administration. This is of translational relevance, since obesity associates to skin inflammatory conditions (55) and also to poor VitD status due to liver dysfunction (56). Thus, sc administration of cholecalciferol under inflammatory environments could assure sufficient 25-hydroxy and 1,25-hydroxy VitD local availability for tolDC-conditioning and Treg induction. Moreover, we consider that inflammation-triggered local activation of cholecalciferol is convenient, since the extent of this activation under non-limiting amounts of the prohormone due to its sc repetitive supply would be proportional to the inflammatory burden, leading to a controlled form of active VitD delivery that matches the tolerogenic requirements. Future work comparing the immunoregulatory capacity of sc low-dose cholecalciferol, 25-hydroxy and active VitD, combined to Dexa, will permit a better understanding of the atheroprotective effects reported here and the development of improved Treg-inducing VitD/Dexa-based antiatherogenic formulations.

Metabolism and immunity are two intimately interconnected processes that are considered to play central role in atherosclerosis pathogenesis and CVD (57, 58). Since signaling through the VDR and GR exerts effects on both processes, it was of interest that no evidence of metabolic changes, such as lipid-lowering effects, associated to the VitD/Dexa-mediated atheroprotection were found (Figure 7 and Table 1). As explained before, the dose, via and scheme of treatments utilized here and our findings of no toxicity or immunosuppression, argues to non-systemic relevant distribution of the agents that could have affected directly the liver or other key metabolic organ. However, FOXP3^+^ Tregs have been shown to influence hepatic metabolism of lipoproteins and control circulating cholesterol levels (59), which raises the question of why our VitD/Dexa treatment did not alter blood lipids indirectly via Treg-promotion. We believe that a more complete analysis of lipoprotein distribution could help to clarify this, or that Tr1-like cells (in which VitD/Dexa impact was more pronounced; Figure 4) are weaker at “lipid-lowering” compared to FOXP3^+^ Treg. Alternatively, taking in account the complexity of this new field of immunometabolism, it is plausible that the magnitude and specificity of the atheroprotective pathways triggered by a particular immunotherapeutic strategy underlies the chances to significantly affect organismal metabolism. Thus, in accordance with our results, a recent work inducing epitope-specific Treg cells via vaccination was atheroprotective without affecting blood lipids (60). Moreover, not only the relevant CANTOS study, but also a number of immunomodulatory interventions in animal models, such as other Treg-inducer (61), a TLR4 inhibitor (62), an antiplatelet agent (63), NLRP3 inflammasome-directed therapies (64, 65) or a M2 macrophage-inducing kinase inhibitor (66) exerted atheroprotection in the absence of lipid-modifying effects, suggesting that lipid-lowering effects of immune-mediated treatments are possibly the exception rather than the rule.

It is tempting to explain the observed VitD/Dexa-induced atheroprotection as a consequence of a better status of VitD and/or direct suppressive effects of Dexa on atheromatous artery. However, we consider this unlikely for the following reasons: (1) this idea implies systemic circulation and distribution of the agents for which as we explained, although not directly proven here, no indirect evidence was found. (2) A better VitD status requires that mice responded to VitD replenishment, which has been demonstrated not to be the case in obese mice (56, 67). (3) Direct atheroprotective activity of glucocorticoids on the atheromatous artery is controversial and not only anti-atherogenic but also pro-atherogenic effects have been reported (68–79). (4) Systemic exposure to Dexa promotes metabolic changes, such as high serum lipids and glucose, and some other alterations, not found here (Table 1). Instead, the vigorous increase in IL-10-producing leukocytes (Supplementary Figure S7) argues in favor of an indirect immunoregulatory-operating process and a rather negligible direct athero-suppressive effect of Dexa or a VitD-improving action in our VitD/Dexa-treated mice as the key contributors to atheroprotection.

One limitation of the present study is that although some immunological insights were presented as likely contributors to the therapeutic effect, definitive experimental evidence of the mechanisms of atheroprotection operating in VitD/Dexa-treated mice was not obtained. Likewise, whether VitD/Dexa treatment inhibits atherosclerosis progression in already established plaques was not investigated here, and both aspects could be of future key value from the translational point of view. This, of course, does not change the value of this preclinical proof of concept for a small molecule formulation that induces Tregs and mitigates chronic inflammation in an extraordinarily relevant pathological scenario like atherosclerosis. Despite the broad clinical interest, no drug is currently available in the clinics specifically intended to induce Tregs. Clinical trials are in course in which the therapeutic potential of low-dose IL-2 is being evaluated as a Treg-inducer for inflammatory diseases, including atherosclerosis (80). Independent of the safety/efficacy outcomes of these trials, it is predictable that the costs of this biological agent will make it unrealistic for the routine medical practice. This contrasts with the low-cost, simplicity and convenience of the VitD/Dexa formulation, which make the two agents excellent candidates for reformulation, repurposing, clinical development and realistic clinical use.

## Conclusion

In conclusion, we demonstrated that a simple combined formulation of two well-known small molecules, administered locally in the footpad via sc route and following a low-dose scheme with the intention to avoid systemic exposure, was able not only to safely promote regulatory immune cells but also to impact atherosclerosis development *in vivo*. This alternative immune intervention combining two cheap “old drugs” could be easily reformulated in a clinically compatible preparation for human proof of principle testing and clinical development. Moreover, we propose this approach as an attractive prophylactic or therapeutic option for other medical conditions in which stimulation of Treg have therapeutic potential, such as autoimmune, allergic, and other chronic inflammatory diseases.

## Data Availability Statement

The datasets generated for this study are available on request to the corresponding author.

## Ethics Statement

The animal study was reviewed and approved by the Comite Institucional para el Uso y Cuidado de los Animales de Experimentacion, Universidad de Antioquia.

## Author Contributions

JR-P conceived, designed and directed the study, and wrote the manuscript. LO-Q, JJ, JT-G, and JR-P designed and planned the experiments. LO-Q, JJ, and JT-G conducted the experiments. LO-Q, JJ, and JR-P analyzed the results and interpreted the data. LO-Q and JJ contributed to manuscript writing and preparation. All authors read and approved the final manuscript.

## Conflict of Interest

The authors declare that the research was conducted in the absence of any commercial or financial relationships that could be construed as a potential conflict of interest.

## Abbreviations

APC: antigen-presenting cell
ApoE^–/–^: apolipoprotein E-deficient
ASA: acetylsalicylic acid
CANTOS: canakinumab anti-inflammatory thrombosis outcomes study
CD: cluster of differentiation
cDC: conventional DC
Curc: curcumin
CVD: cardiovascular diseases
Dexa: dexamethasone
dLN: draining lymph node
FACS: fluorescence-activated cell sorting
FOXP3: forkhead box P3
GR: glucocorticoid receptor
HFD: high-fat diet
IFN γ: interferon gamma
Ig: immunoglobulin
IL: interleukin
LDL: low-density lipoproteins
MDA: malondialdehyde
MDSC: myeloid derived suppressor cells
MFI: mean fluorescence intensity
MHC: major histocompatibility complex
MLN: mediastinal lymph node
MoDC: monocyte-derived dendritic cells
NAC: N-acetyl cysteine
NK: natural killer
NR: nuclear receptors
pDC: plasmacytoid dendritic cells
PLN: popliteal lymph node
Querc: quercetin
rapa: rapamycin
sc: subcutaneous
SP: spleen
Teff: effector T cells
Th1: type 1 T helper
tolDC: tolerogenic dendritic cells
Tr1: type 1 regulatory T cell
Treg: regulatory T cells
VDR: vitamin D receptor
Vit: vitamin
VitD: vitamin D3
WHO: world health organization.

## References

[1] Herrington W Lacey B Sherliker P Armitage J Lewington S. Epidemiology of atherosclerosis and the potential to reduce the global burden of Atherothrombotic disease. *Circ Res.* (2016) 118:535–46. 10.1161/CIRCRESAHA.115.307611 26892956

[2] Libby P Bornfeldt KE Tall AR. Atherosclerosis: successes, surprises, and future challenges. *Circ Res.* (2016) 118:531–4. 10.1161/CIRCRESAHA.116.308334 26892955 PMC4762065

[3] McClellan M Brown N Califf RM Warner JJ. Call to action: urgent challenges in Cardiovascular disease: a presidential advisory from the American heart association. *Circulation.* (2019) 139:E44–54. 10.1161/CIR.0000000000000652 30674212

[4] Libby P Lichtman AH Hansson GK. Immune effector mechanisms implicated in atherosclerosis: from mice to humans. *Immunity.* (2013) 38:1092–104. 10.1016/j.immuni.2013.06.009 23809160 PMC3764500

[5] Tabas I Lichtman AH. Monocyte-macrophages and T cells in atherosclerosis. *Immunity.* (2017) 47:621–34. 10.1016/j.immuni.2017.09.008 29045897 PMC5747297

[6] Gisterå A Hansson GK. The immunology of atherosclerosis. *Nat Rev Nephrol.* (2017) 13:368–80. 10.1038/nrneph.2017.51 28392564

[7] Welsh P Grassia G Botha S Sattar N Maffia P. Targeting inflammation to reduce cardiovascular disease risk: a realistic clinical prospect? *Br J Pharmacol.* (2017) 174:3898–913. 10.1111/bph.13818 28409825 PMC5660005

[8] Ridker PM Everett BM Thuren T MacFadyen JG Chang WH Ballantyne C Antiinflammatory therapy with canakinumab for Atherosclerotic disease. *N Engl J Med.* (2017) 377:1119–31. 10.1056/NEJMoa1707914 28845751

[9] Hansson GK. Inflammation and atherosclerosis: the end of a controversy. *Circulation.* (2017) 136:1875–7. 10.1161/CIRCULATIONAHA.117.030484 28916641

[10] Lutgens E Atzler D Döring Y Duchene J Steffens S Weber C. Immunotherapy for cardiovascular disease. *Eur Heart J.* (2019) 40:3937–46. 10.1093/eurheartj/ehz283 31121017

[11] Nilsson J Lichtman A Tedgui A. Atheroprotective immunity and cardiovascular disease: therapeutic opportunities and challenges. *J Intern Med.* (2015) 278:507–19. 10.1111/joim.12353 25659809

[12] Foks AC Lichtman AH Kuiper J. treating atherosclerosis with regulatory T cells. *Arterioscler Thromb Vasc Biol.* (2015) 35:280–7. 10.1161/ATVBAHA.114.303568 25414253 PMC4715365

[13] Meng X Yang J Dong M Zhang K Tu E Gao Q Regulatory T cells in cardiovascular diseases. *Nat Rev Cardiol.* (2016) 13:167–79. 10.1038/nrcardio.2015.169 26525543 PMC11849084

[14] Sage AP Mallat Z. Readapting the adaptive immune response – therapeutic strategies for atherosclerosis. *Br J Pharmacol.* (2017) 174:3926–39. 10.1111/bph.13700 28052311 PMC5659986

[15] Serra P Santamaria P. Antigen-specific therapeutic approaches for autoimmunity. *Nat Biotechnol.* (2019) 37:238–51. 10.1038/s41587-019-0015-4 30804535

[16] Hackstein H Thomson AW. Dendritic cells: emerging pharmacological targets of immunosuppressive drugs. *Nat Rev Immunol.* (2004) 4:24–35. 10.1038/nri1256 14704765

[17] Tan PH Sagoo P Chan C Yates JB Campbell J Beutelspacher SC Inhibition of NF-κB and oxidative pathways in human dendritic cells by antioxidative vitamins generates regulatory T cells. *J Immunol.* (2005) 174:7633–44. 10.4049/jimmunol.174.12.7633 15944264

[18] Huang R-Y Yu Y-L Cheng W-C OuYang C-N Fu E Chu C-L. Immunosuppressive effect of quercetin on dendritic cell activation and function. *J Immunol.* (2010) 184:6815–21. 10.4049/jimmunol.0903991 20483746

[19] Cong Y Wang L Konrad A Schoeb T Elson CO. Curcumin induces the tolerogenic dendritic cell that promotes differentiation of intestine-protective regulatory T cells. *Eur J Immunol.* (2009) 39:3134–46. 10.1002/eji.200939052 19839007

[20] Buckland M Jago C Fazekesova H George A Lechler R Lombardi G. Aspirin modified dendritic cells are potent inducers of allo-specific regulatory T-cells. *Int Immunopharmacol.* (2006) 6:1895–901. 10.1016/j.intimp.2006.07.008 17219690

[21] Kang Y Xu L Wang B Chen A Zheng G. Cutting edge: immunosuppressant as adjuvant for tolerogenic immunization. *J Immunol.* (2008) 180:5172–6. 10.4049/jimmunol.180.8.5172 18390698 PMC2377418

[22] Lara-Guzman OJ Tabares-Guevara JH Leon-Varela YM Álvarez RM Roldan M Sierra JA Proatherogenic macrophage activities are targeted by the flavonoid quercetin. *J Pharmacol Exp Ther.* (2012) 343:296–306. 10.1124/jpet.112.196147 22869926

[23] Kalra N Ishmael FT. Cross-talk between vitamin D, estrogen and corticosteroids in glucocorticoid resistant asthma. *OA Inflamm.* (2014) 2:2.

[24] Wöbke TK Sorg BL Steinhilber D. Vitamin D in inflammatory diseases. *Front Physiol.* (2014) 5:244. 10.3389/fphys.2014.00244 25071589 PMC4078458

[25] Litonjua AA. Vitamin D and corticosteroids in asthma: synergy, interaction and potential therapeutic effects. *Expert Rev Respir Med.* (2013) 7:101–4. 10.1586/ers.12.85 23547985

[26] Nikolic T Roep BO. Regulatory multitasking of tolerogenic dendritic cells – lessons taken from vitamin D3-treated tolerogenic dendritic cells. *Front Immunol.* (2013) 4:113. 10.3389/fimmu.2013.00113 23717310 PMC3653108

[27] Gordon JR Ma Y Churchman L Gordon SA Dawicki W. Regulatory dendritic cells for immunotherapy in immunologic diseases. *Front Immunol.* (2014) 5:7. 10.3389/fimmu.2014.00007 24550907 PMC3907717

[28] Cochain C Zernecke A. Protective and pathogenic roles of CD8+ T cells in atherosclerosis. *Basic Res Cardiol.* (2016) 111:71. 10.1007/s00395-016-0589-7 27783202

[29] Massy ZA Ivanovski O Nguyen-Khoa T Angulo J Szumilak D Mothu N Uremia accelerates both atherosclerosis and arterial calcification in apolipoprotein E knockout mice. *J Am Soc Nephrol.* (2005) 16:109–16. 10.1681/ASN.2004060495 15563564

[30] Tabas I Glass CK. Anti-inflammatory therapy in chronic disease: challenges and opportunities. *Science.* (2013) 339:166–72. 10.1126/science.1230720 23307734 PMC3608517

[31] Singh JA Wells GA Christensen R Tanjong Ghogomu E Maxwell LJ MacDonald JK Adverse effects of biologics: a network meta-analysis and Cochrane overview. *Cochrane Database Syst Rev.* (2012) 2011:CD008794. 10.1002/14651858.CD008794 21328309 PMC7173749

[32] Bonow RO Harrington RA Yancy CW. Cost-effectiveness of PCSK9 Inhibitors. *JAMA Cardiol.* (2017) 2:1298–9. 10.1001/jamacardio.2017.3656 29049827

[33] Sehested TSG Bjerre J Ku S Chang A Jahansouz A Owens DK Cost-effectiveness of Canakinumab for prevention of recurrent cardiovascular events. *JAMA Cardiol.* (2019) 4:128–35. 10.1001/jamacardio.2018.4566 30649147 PMC6439626

[34] Mosanya CH Isaacs JD. Tolerising cellular therapies: what is their promise for autoimmune disease? *Ann Rheum Dis.* (2019) 78:297–310. 10.1136/annrheumdis-2018-214024 30389690 PMC6390030

[35] Christakos S Dhawan P Verstuyf A Verlinden L Carmeliet G. Vitamin D: metabolism, molecular mechanism of action, and pleiotropic effects. *Physiol Rev.* (2016) 96:365–408. 10.1152/physrev.00014.2015 26681795 PMC4839493

[36] Cari L De Rosa F Nocentini G Riccardi C. Context-dependent effect of glucocorticoids on the proliferation, differentiation, and apoptosis of regulatory T cells: a review of the empirical evidence and clinical applications. *Int J Mol Sci.* (2019) 20:1142. 10.3390/ijms20051142 30845709 PMC6429178

[37] Burris TP Solt LA Wang Y Crumbley C Banerjee S Griffett K Nuclear receptors and their selective pharmacologic modulators. *Pharmacol Rev.* (2013) 65:710–78. 10.1124/pr.112.006833 23457206 PMC11060414

[38] Chen A Geng Y Ke H Constant L Yan Z Pan Y Cutting edge: dexamethasone potentiates the responses of both regulatory T cells and B-1 cells to antigen immunization in the ApoE -/- mouse model of atherosclerosis. *J Immunol.* (2014) 193:35–9. 10.4049/jimmunol.1302469 24899497 PMC4153946

[39] Takeda M Yamashita T Sasaki N Nakajima K Kita T Shinohara M Oral administration of an active form of vitamin D_3_ (Calcitriol) decreases atherosclerosis in mice by inducing regulatory T cells and immature dendritic cells With tolerogenic functions. *Arterioscler Thromb Vasc Biol.* (2010) 30:2495–503. 10.1161/ATVBAHA.110.215459 20930170

[40] Wigren M Bengtsson D Duneìr P Olofsson K Björkbacka H Bengtsson E Atheroprotective effects of alum are associated with capture of oxidized LDL antigens and activation of regulatory T cells. *Circ Res.* (2009) 104:e62–70. 10.1161/CIRCRESAHA.109.196667 19478203

[41] Hermansson A Johansson DK Ketelhuth DFJ Andersson J Zhou X Hansson GK. Immunotherapy with tolerogenic apolipoprotein B-100–loaded dendritic cells attenuates atherosclerosis in hypercholesterolemic mice. *Circulation.* (2011) 123:1083–91. 10.1161/CIRCULATIONAHA.110.973222 21357823

[42] Mallat Z Gojova A Brun V Esposito B Fournier N Cottrez F Induction of a regulatory T Cell Type 1 response reduces the development of atherosclerosis in apolipoprotein E–knockout mice. *Circulation.* (2003) 108:1232–7. 10.1161/01.CIR.0000089083.61317.A1 12912803

[43] Yun TJ Lee JS Machmach K Shim D Choi J Wi YJ Indoleamine 2,3-dioxygenase-expressing aortic plasmacytoid dendritic cells protect against atherosclerosis by induction of regulatory T cells. *Cell Metab.* (2016) 23:852–66. 10.1016/j.cmet.2016.04.010 27166946

[44] Choi J-H Cheong C Dandamudi DB Park CG Rodriguez A Mehandru S Flt3 signaling-dependent dendritic cells protect against atherosclerosis. *Immunity.* (2011) 35:819–31. 10.1016/j.immuni.2011.09.014 22078798

[45] Zheng G Zhong S Geng Y Munirathinam G Cha I Reardon C Dexamethasone promotes tolerance in vivo by enriching CD11clo CD40lo tolerogenic macrophages. *Eur J Immunol.* (2013) 43:219–27. 10.1002/eji.201242468 23001956 PMC3697049

[46] Sage AP Tsiantoulas D Binder CJ Mallat Z. The role of B cells in atherosclerosis. *Nat Rev Cardiol.* (2019) 16:180–96. 10.1038/s41569-018-0106-9 30410107

[47] Foks AC Van Puijvelde GHM Wolbert J Kröner MJ Frodermann V Van Der Heijden T CD11b^+^ Gr-1^+^ myeloid-derived suppressor cells reduce atherosclerotic lesion development in LDLr deficient mice. *Cardiovasc Res.* (2016) 111:252–61. 10.1093/cvr/cvw114 27234908

[48] Gravano DM Vignali DAA. The battle against immunopathology: infectious tolerance mediated by regulatory T cells. *Cell Mol Life Sci.* (2012) 69:1997–2008. 10.1007/s00018-011-0907-z 22205213 PMC3353028

[49] Zhang Y Li Q Rao E Sun Y Grossmann ME Morris RJ Epidermal fatty acid binding protein promotes skin inflammation induced by high-fat diet. *Immunity.* (2015) 42:953–64. 10.1016/j.immuni.2015.04.016 25992864 PMC4440244

[50] Proto JD Doran AC Gusarova G Yurdagul A Sozen E Subramanian M Regulatory T cells promote macrophage efferocytosis during inflammation resolution. *Immunity.* (2018) 49:666–77. 10.1016/j.immuni.2018.07.015 30291029 PMC6192849

[51] Bäck M Yurdagul A Tabas I Öörni K Kovanen PT. Inflammation and its resolution in atherosclerosis: mediators and therapeutic opportunities. *Nat Rev Cardiol.* (2019) 16:389–406. 10.1038/s41569-019-0169-2 30846875 PMC6727648

[52] MacRitchie N Grassia G Noonan J Cole JE Hughes CE Schroeder J The aorta can act as a site of naïve CD4^+^ T-cell priming. *Cardiovasc Res.* (2019) 116:306–16. 10.1093/cvr/cvz102 30980670

[53] Bscheider M Butcher EC. Vitamin D immunoregulation through dendritic cells. *Immunology.* (2016) 148:227–36. 10.1111/imm.12610 27040466 PMC4913286

[54] Sigmundsdottir H Pan J Debes GF Alt C Habtezion A Soler D DCs metabolize sunlight-induced vitamin D3 to “program” T cell attraction to the epidermal chemokine CCL27. *Nat Immunol.* (2007) 8:285–93. 10.1038/ni1433 17259988

[55] Hirt PA Castillo DE Yosipovitch G Keri JE. Skin changes in the obese patient. *J Am Acad Dermatol.* (2019) 81:1037–57. 10.1016/j.jaad.2018.12.070 31610857

[56] Pourshahidi LK. Vitamin D and obesity: current perspectives and future directions. *Proc Nutr Soc.* (2015) 74:115–24. 10.1017/S0029665114001578 25359323

[57] Getz GS Reardon CA. The mutual interplay of lipid metabolism and the cells of the immune system in relation to atherosclerosis. *Clin Lipidol.* (2014) 9:657–71. 10.2217/clp.14.50 25705263 PMC4335315

[58] Ketelhuth DFJ Lutgens E Bäck M Binder CJ Van den Bossche J Daniel C Immunometabolism and atherosclerosis: perspectives and clinical significance: a position paper from the working group on atherosclerosis and vascular biology of the european society of cardiology. *Cardiovasc Res.* (2019) 115:1385–92. 10.1093/cvr/cvz166 31228191 PMC6681176

[59] Klingenberg R Gerdes N Badeau RM Gisterå A Strodthoff D Ketelhuth DFJ Depletion of FOXP3^+^ regulatory T cells promotes hypercholesterolemia and atherosclerosis. *J Clin Invest.* (2013) 123:1323–34. 10.1172/JCI63891 23426179 PMC3582120

[60] Kimura T Kobiyama K Winkels H Tse K Miller J Vassallo M Regulatory CD4^+^ T cells recognize MHC-II-restricted peptide epitopes of apolipoprotein B. *Circulation.* (2018) 138:1130–43. 10.1161/CIRCULATIONAHA.117.031420 29588316 PMC6160361

[61] Ji Q Meng K Yu K Huang S Huang Y Min X Exogenous interleukin 37 ameliorates atherosclerosis via inducing the Treg response in ApoE-deficient mice. *Sci Rep.* (2017) 7:3310. 10.1038/s41598-017-02987-4 28607385 PMC5468328

[62] Wang X-Q Wan H-Q Wei X-J Zhang Y Qu P. CLI-095 decreases atherosclerosis by modulating foam cell formation in apolipoprotein E-deficient mice. *Mol Med Rep.* (2016) 14:49–56. 10.3892/mmr.2016.5233 27176130 PMC4918599

[63] Heim C Gebhardt J Ramsperger-Gleixner M Jacobi J Weyand M Ensminger SM. Clopidogrel significantly lowers the development of atherosclerosis in ApoE-deficient mice in vivo. *Heart Vessels.* (2016) 31:783–94. 10.1007/s00380-015-0696-7 26062773

[64] Çimen I Kocatürk B Koyuncu S Tufanlı Ö Onat UI Yıldırım AD Prevention of atherosclerosis by bioactive palmitoleate through suppression of organelle stress and inflammasome activation. *Sci Transl Med.* (2016) 8:358ra126. 10.1126/scitranslmed.aaf9087 27683551

[65] Zhang R Han S Zhang Z Zhang W Yang J Wan Z Cereal fiber ameliorates high-fat/cholesterol-diet-induced atherosclerosis by modulating the NLRP3 inflammasome pathway in ApoE ^–/–^ Mice. *J Agric Food Chem.* (2018) 66:4827–34. 10.1021/acs.jafc.8b00380 29664631

[66] Wang Z Wang S Wang Z Yun T Wang C Wang H. Tofacitinib ameliorates atherosclerosis and reduces foam cell formation in apoE deficient mice. *Biochem Biophys Res Commun.* (2017) 490:194–201. 10.1016/j.bbrc.2017.06.020 28601639

[67] Roizen JD Long C Casella A O’Lear L Caplan I Lai M Obesity decreases hepatic 25-hydroxylase activity causing low serum 25-hydroxyvitamin D. *J Bone Miner Res.* (2019) 34:1068–73. 10.1002/jbmr.3686 30790351 PMC6663580

[68] Yang L Yang JB Chen J Yu GY Zhou P Lei L Enhancement of human ACAT1 gene expression to promote the macrophage-derived foam cell formation by dexamethasone. *Cell Res.* (2004) 14:315–23. 10.1038/sj.cr.7290231 15353128

[69] Hirsch LJ Mazzone T. Dexamethasone modulates lipoprotein metabolism in cultured human monocyte-derived macrophages. Stimulation of scavenger receptor activity. *J Clin Invest.* (1986) 77:485–90. 10.1172/JCI112327 3944266 PMC423369

[70] Poon M Gertz SD Fallon JT Wiegman P Berman JW Sarembock IJ Dexamethasone inhibits macrophage accumulation after balloon arterial injury in cholesterol fed rabbits. *Atherosclerosis.* (2001) 155:371–80. 10.1016/S0021-9150(00)00605-5 11254907

[71] Hagihara H Nomoto A Mutoh S Yamaguchi I Ono T. Role of inflammatory responses in initiation of atherosclerosis: effects of anti-inflammatory drugs on cuff-induced leukocyte accumulation and intimal thickening of rabbit carotid artery. *Atherosclerosis.* (1991) 91:107–16. 10.1016/0021-9150(91)90192-6 1811546

[72] Ayaori M Sawada S Yonemura A Iwamoto N Ogura M Tanaka N Glucocorticoid receptor regulates ATP-binding cassette transporter-A1 expression and apolipoprotein-mediated cholesterol efflux from macrophages. *Arterioscler Thromb Vasc Biol.* (2006) 26:163–8. 10.1161/01.ATV.0000193513.29074.52 16254209

[73] Nashel DJ. Is atherosclerosis a complication of long-term corticosteroid treatment? *Am J Med.* (1986) 80:925–9. 10.1016/0002-9343(86)90639-X 3518440

[74] Naito M Yasue M Asai K Yamada K Hayashi T Kuzuya M Effects of dexamethasone on experimental atherosclerosis in cholesterol-fed rabbits. *J Nutr Sci Vitaminol (Tokyo).* (1992) 38:255–64. 10.3177/jnsv.38.255 1453236

[75] Asai K Funaki C Hayashi T Yamada K Naito M Kuzuya M Dexamethasone-induced suppression of aortic atherosclerosis in cholesterol-fed rabbits. Possible mechanisms. *Arterioscler Thromb Vasc Biol.* (1993) 13:892–9. 10.1161/01.ATV.13.6.892 8499410

[76] Zhang W Wang X Hu W Liu L Li X Han J Co-treatment of pitavastatin and dexamethasone exacerbates the high-fat diet–induced atherosclerosis in apoE-deficient mice. *J Cardiovasc Pharmacol.* (2015) 66:189–95. 10.1097/FJC.0000000000000264 25874855

[77] Tous M Ribas V Escolà-Gil JC Blanco-Vaca F Calpe-Berdiel L Coll B Manipulation of inflammation modulates hyperlipidemia in apolipoprotein E-deficient mice: a possible role for interleukin-6. *Cytokine.* (2006) 34:224–32. 10.1016/j.cyto.2006.05.007 16815711

[78] Cheng W Kvilekval KV Abumrad NA. Dexamethasone enhances accumulation of cholesteryl esters by human macrophages. *Am J Physiol Metab.* (1995) 269:E642–8. 10.1152/ajpendo.1995.269.4.E642 7485476

[79] Hirosumi J Nomoto A Ohkubo Y Sekiguchi C Mutoh S Yamaguchi I Inflammatory responses in cuff-induced atherosclerosis in rabbits. *Atherosclerosis.* (1987) 64:243–54. 10.1016/0021-9150(87)90252-8 3606722

[80] Zhao TX Mallat Z. Targeting the immune system in atherosclerosis. *J Am Coll Cardiol.* (2019) 73:1691–706. 10.1016/j.jacc.2018.12.083 30947923

