# Supplementary material for: Reformulating Small Molecules for Cardiovascular Disease Immune Intervention: Low-Dose Combined Vitamin D/Dexamethasone Promotes IL-10 Production and Atheroprotection in Dyslipidemic Mice
Source: Front Immunol. 2020 Apr 24;11:743. doi: 10.3389/fimmu.2020.00743 (PMC7197409; doi:10.3389/fimmu.2020.00743)
Supplement: Supplementary file 1 [file Data_Sheet_1.zip › Data sheet 1 with corrections.docx]

# Supplementary Material

**Supplementary Figures**

**
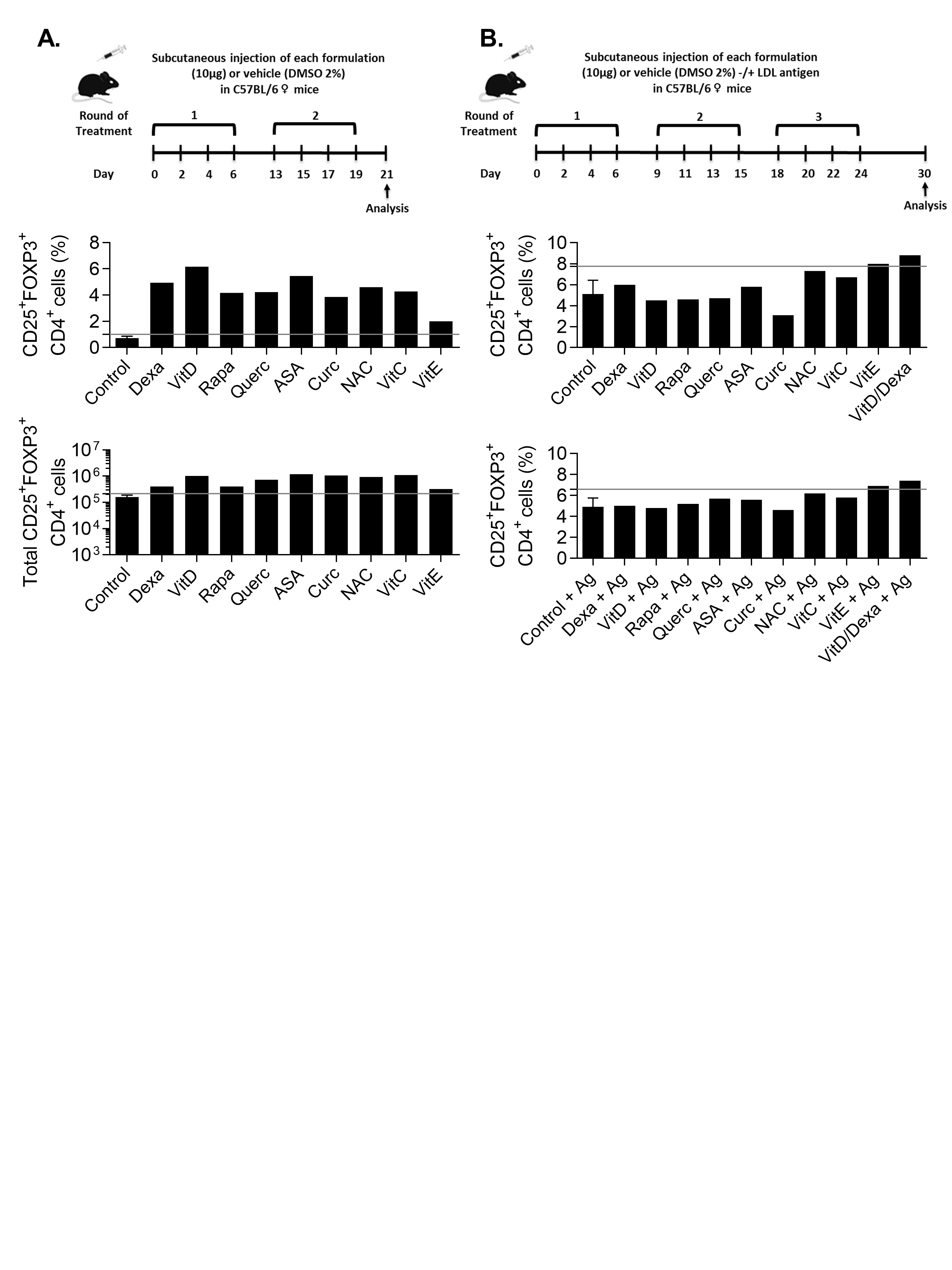
**

**Supplementary Figure S1. Pilot screening of Treg-inducing adjuvants *in vivo*.** A collection of small molecules with documented Treg-inducing capacity was tested in an *in vivo* assay. 7-8 weeks old female C57BL/6 mice were treated sc in the footpad with 10 µg of the indicated compound on the days 0, 2, 4 and 6 to form a round of treatment, as shown (A). Control animals were injected with a similar volume of vehicle (2% DMSO in PBS). After two rounds of treatment separated by a week, animals were sacrificed and the percentage and total number of splenic CD25^+^ FOXP3^+^ CD4^+^ T cells (**Supplementary Figure S3B**) were determined. In a second experiment, 8-12 weeks old female mice received similar sc rounds of 10 µg adjuvant, but, this time: i) three rounds were used, ii) intervals between rounds were shortened to 3 days, and iii) parallel groups of mice were treated with the same amount of adjuvant in combination of the atheroantigen LDL (a mix of LDL/oxLDL 5 µg/5 µg) on the days 4 and 6 of each round, and iv) one more condition (VitD/Dexa, 10 µg each) was included (B). The amounts of splenic CD25^+^ FOXP3^+^ CD4^+^ T cells were then determined by flow cytometry and graphed as percentages in the two separate groups (injected with adjuvant alone, top; or with adjuvant and atheroantigen, bottom; B). The bars of the control groups represent the mean±SD (5-6 mice). The treatment groups consisted of a single mouse per treatment and bars show the result. The grey lines indicate the mean+2SD of the control group as a cut-off. Dexa: Dexamethasone, Rapa: Rapamycin, ASA: Acetylsalicylic acid, Querc: Quercetin, Curc: Curcumin, NAC: *N*-acetylcysteine, VitC: Vitamin C, VitE: Vitamin E and VitD: Vitamin D. Ag: LDL/oxLDL antigen. Note that under the conditions of the first screening, a significant Treg-inducing capacity was confirmed for all compounds (A), whereas under the conditions of the second screening, the combined VitD/Dexa formulation exhibited the highest potency both in the absence or the presence of atheroantigen (B).

**
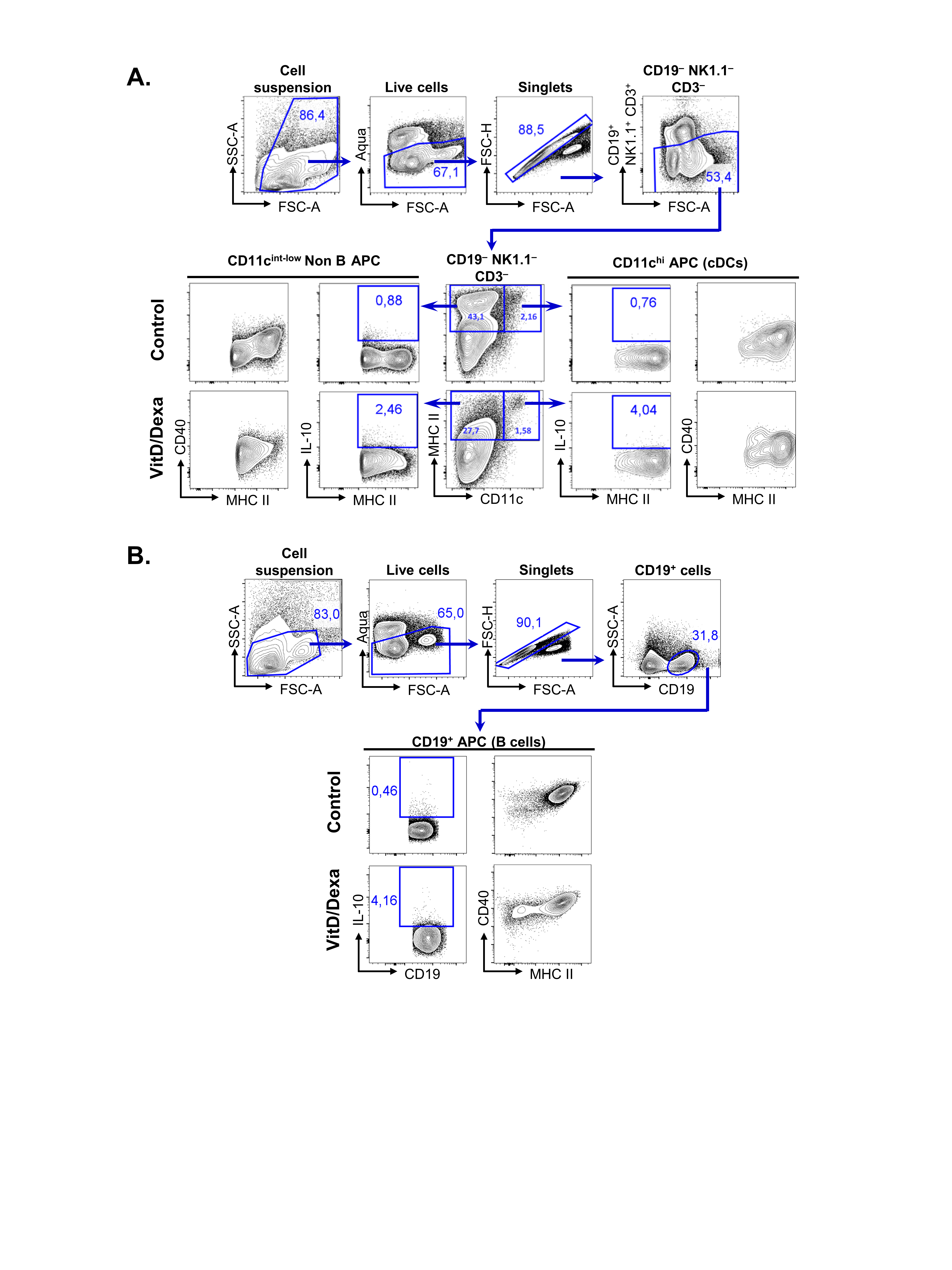
**

**Supplementary Figure S2. Flow cytometric gating strategy for APC analysis.** From the spleen (SP) and popliteal lymph nodes (PLN) cell suspensions, dead cells, doublets, CD19^+^, NK1.1^+^ and CD3^+^ cells were excluded from analysis (A). Gated cells were classified as CD11c^hi^ MHC-II^+^ cDCs or CD11c^int-low^ MHC-II^+^ non-B APC according to the expression of CD11c and MHC-II. For B cell analysis, live cells, singlets, CD19^+^ cells were also selected (B). Finally, IL-10 expression as well as the surface density of MHC-II and CD40 were analysed (A, B).


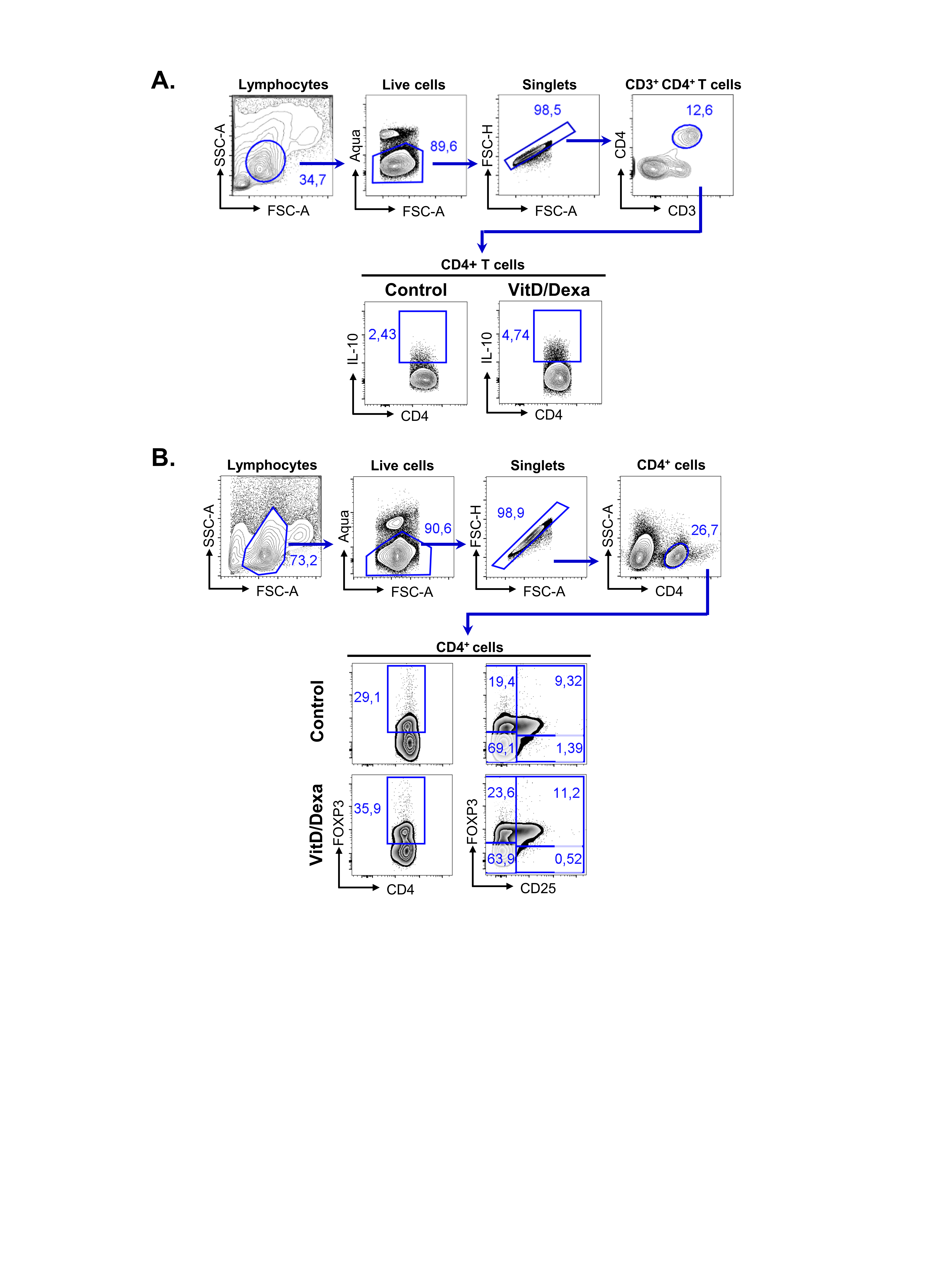


**Supplementary Figure S3. Flow cytometric gating strategy for the analysis of IL-10^+^ CD4^+^ T cells and FOXP3^+^ CD4^+^ regulatory T cells.** From the SP, PLN and mediastinal lymph nodes (MLN) cell suspensions, lymphoid, live, singlets, CD3^+^ CD4^+^ cells were examined for the expression of IL-10 (A). Lymphoid live singlet CD4^+^ cells were also examined for the expression of CD25 and forkhead box P3 (FOXP3) (B). Percentages and total numbers of IL-10- or FOXP3-expressing CD4 cells, as well as the FOXP3 mean of fluorescent intensity (MFI), were analysed.





**Supplementary Figure S4. Flow cytometric gating strategy for the analysis of IFNγ^+^ CD4^+^ T cells and IFNγ^+^ CD8^+^ T cells.** Splenic lymphoid live singlet CD3^+^ CD4^+^ (A) or CD3^+^ CD8^+^ (B) T cells were examined for the expression of IFNγ.


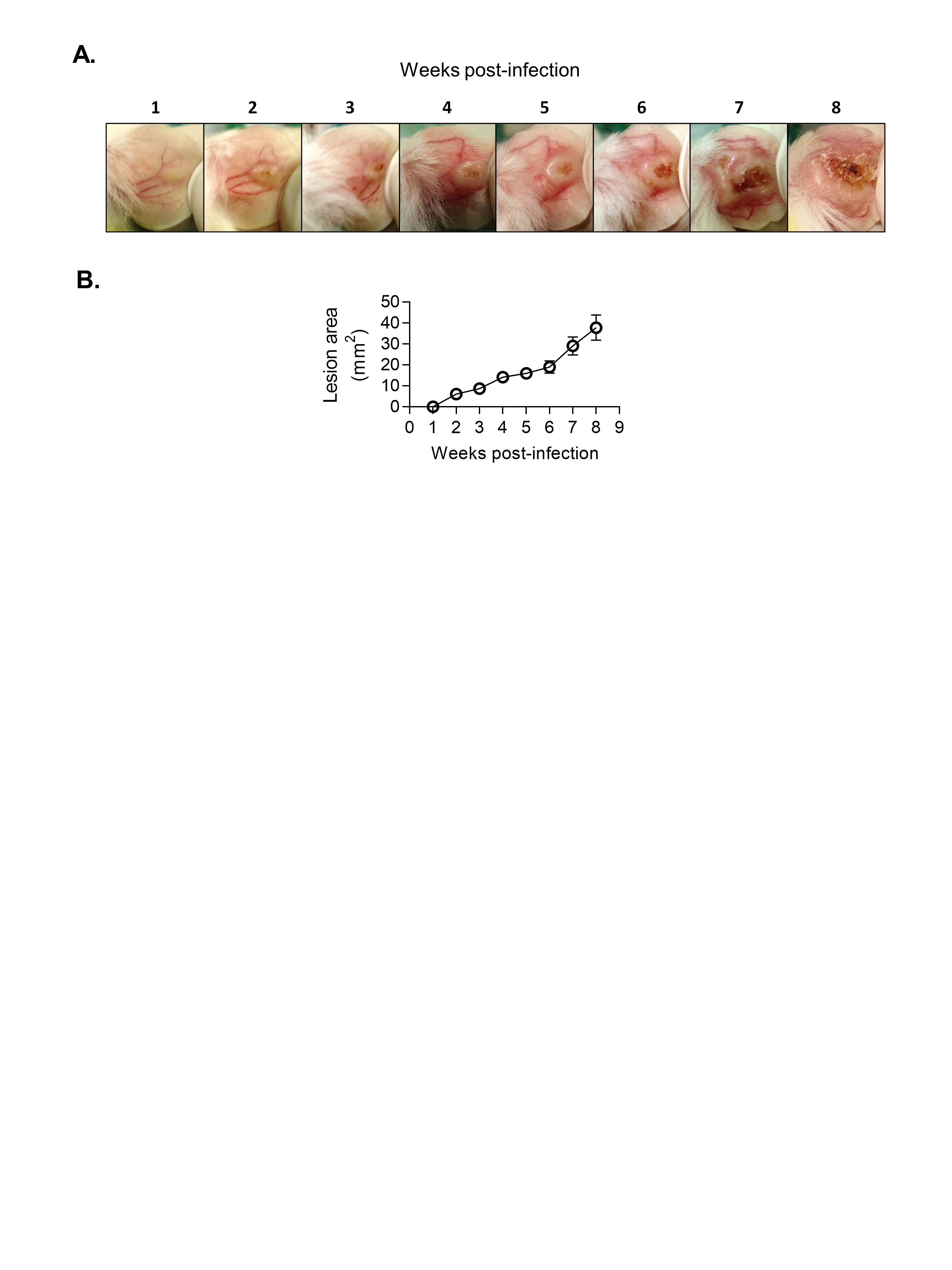


**Supplementary Figure S5. Lesion development after an infective inoculum with *Leishmania panamensis* parasites in BALB/c mice.** Female BALB/6 mice were infected i.d. in the ear with 1x10^5^ stationary-phase *Leishmania panamensis* promastigotes and disease development monitored weekly. Representative photographs from infected ears are shown at different time points post-infection (A). Lesion size was also measured and reported as the area in mm^2^ (B). Note the growth of the lesions that progress to ulcerative forms compromising almost the entire ear. Data are presented as mean±SEM (8 mice).

**
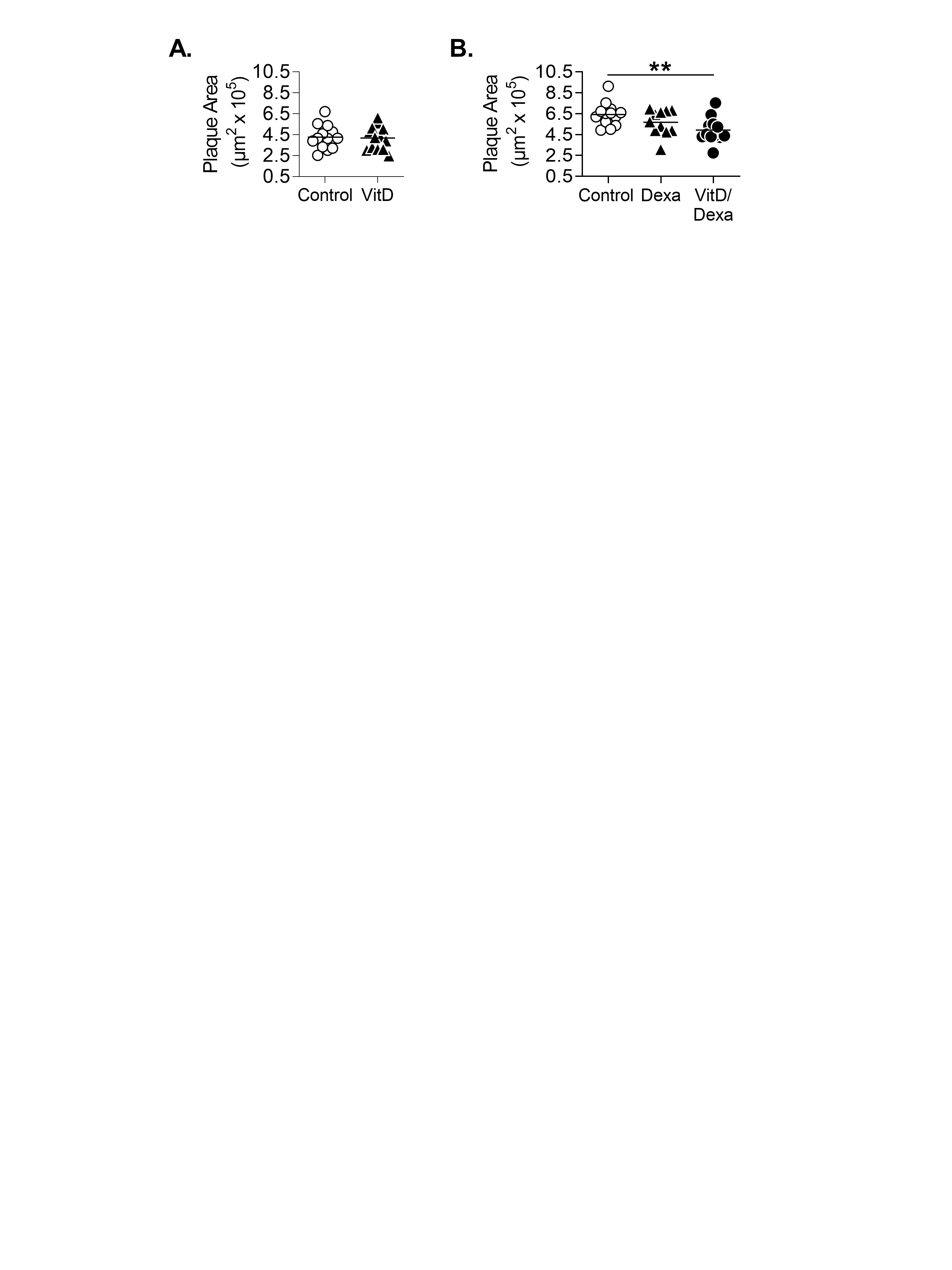
**

**Supplementary Figure S6. Significant atheroprotection by VitD and Dexa requires the combined formulation.** 8-12 weeks old male ApoE^-/-^ mice were treated sc with VitD (10 μg) or vehicle and fed a HFD, as shown in **Figure 1** (A). A similar protocol was used in an independent experiment in which groups of 11-17 weeks old female ApoE^-/-^ mice were treated sc with Dexa (10 μg), VitD/Dexa (10 μg/10 μg) or vehicle (B). After sacrifice, cryosections from the aortic root were stained with H/E and the areas of the atheromatous lesions were calculated with image analysis software. Each point represents the average area per mouse (7-9 sections/mouse), and bars represent the mean. **P<0.01 (One-way ANOVA test followed by Bonferroni’s post-test). Note that no atheroprotective effect is induced by VitD alone (A) and a non-significant atheroprotective trend is induced by Dexa (B), whereas the combined formulation VitD/Dexa induced significant atheroprotection in female ApoE^-/-^ mice (B) as it was observed in male ApoE^-/-^ mice (**Figure 7**).


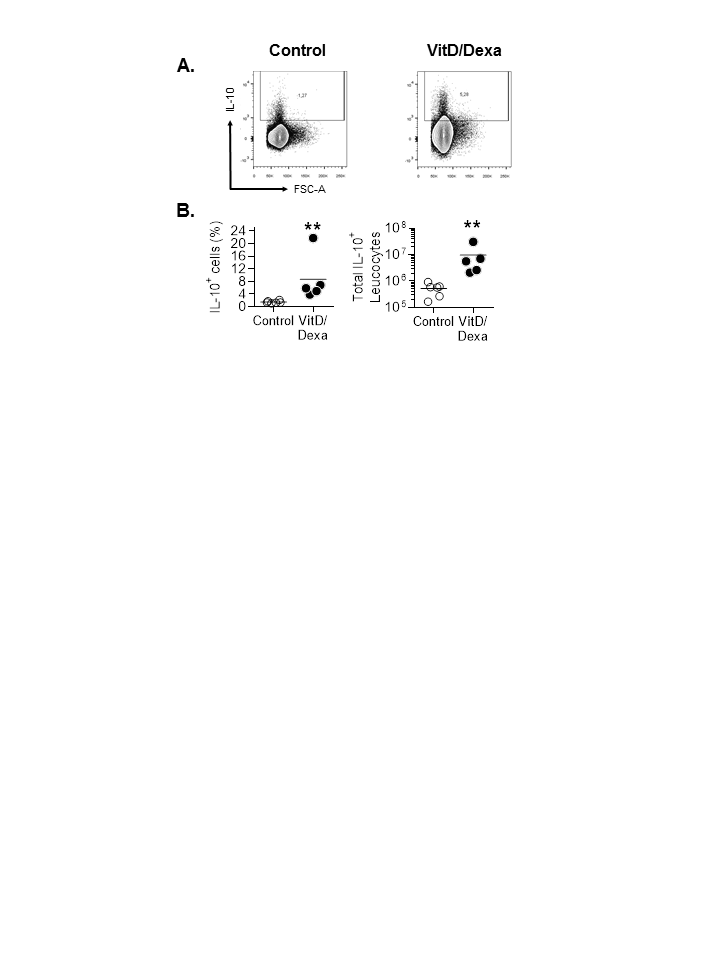


**Supplementary Figure S7. IL-10 production by bulk splenocytes.** Total leucocyte suspensions from the SP of VitD/Dexa- or vehicle-treated mice stimulated with PMA/ionomycin were stained and gated to select singlet live total leucocytes (as in **Supplementary Figure S2**, but with no further gating) and the amounts of IL-10-producing cells determined. Representative dot plots are presented (A). The percentages (left) and absolute numbers (right) of IL-10-producing leucocytes are shown (B). Lines represent the mean from 5-6 mice/group. **P<0.01 (Mann Whitney test).


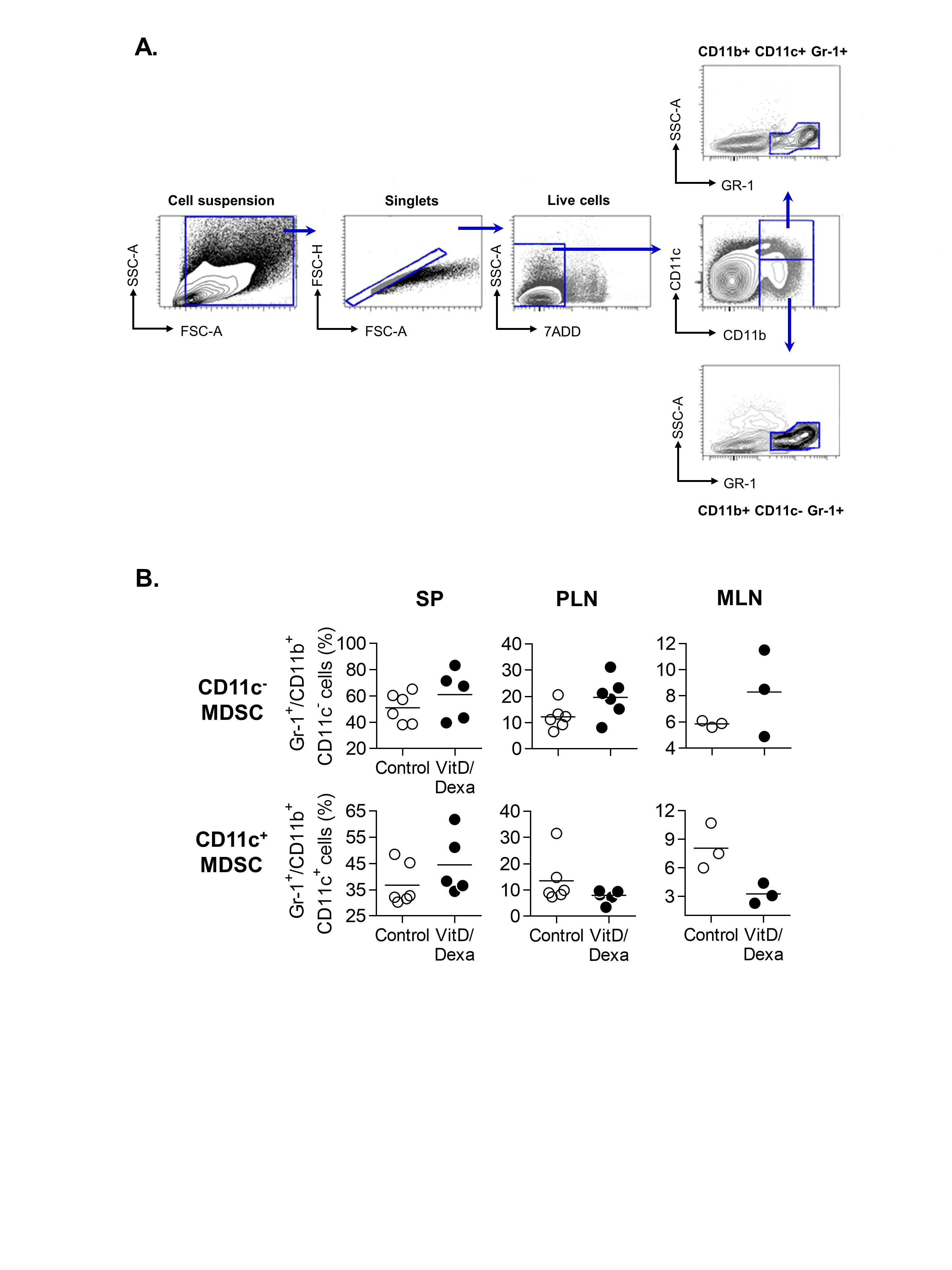


**Supplementary Figure S8. No significant increase in MDSC from VitD/Dexa-treated mice**. PMA/ionomycin-stimulated SP, PLN and MLN cell suspensions from VitD/Dexa- or vehicle-treated mice were stained and gated to discriminate CD11b^+^ CD11c^-^ Gr1^+^ MDSC cells (A). A CD11c^+^ MDSC population was also considered, as suggested by some authors (1), by defining the gate of live CD11b^+^ CD11c^+^ Gr1^+^ cells, as shown (A). The percentages of the indicated MDSC was calculated in individual mice and graphed (B). The bars indicate the mean, from 3-6 mice/group. No significant increase in the frequency of MDSC was observed in all lymphoid organs (Mann Whitney test).

**
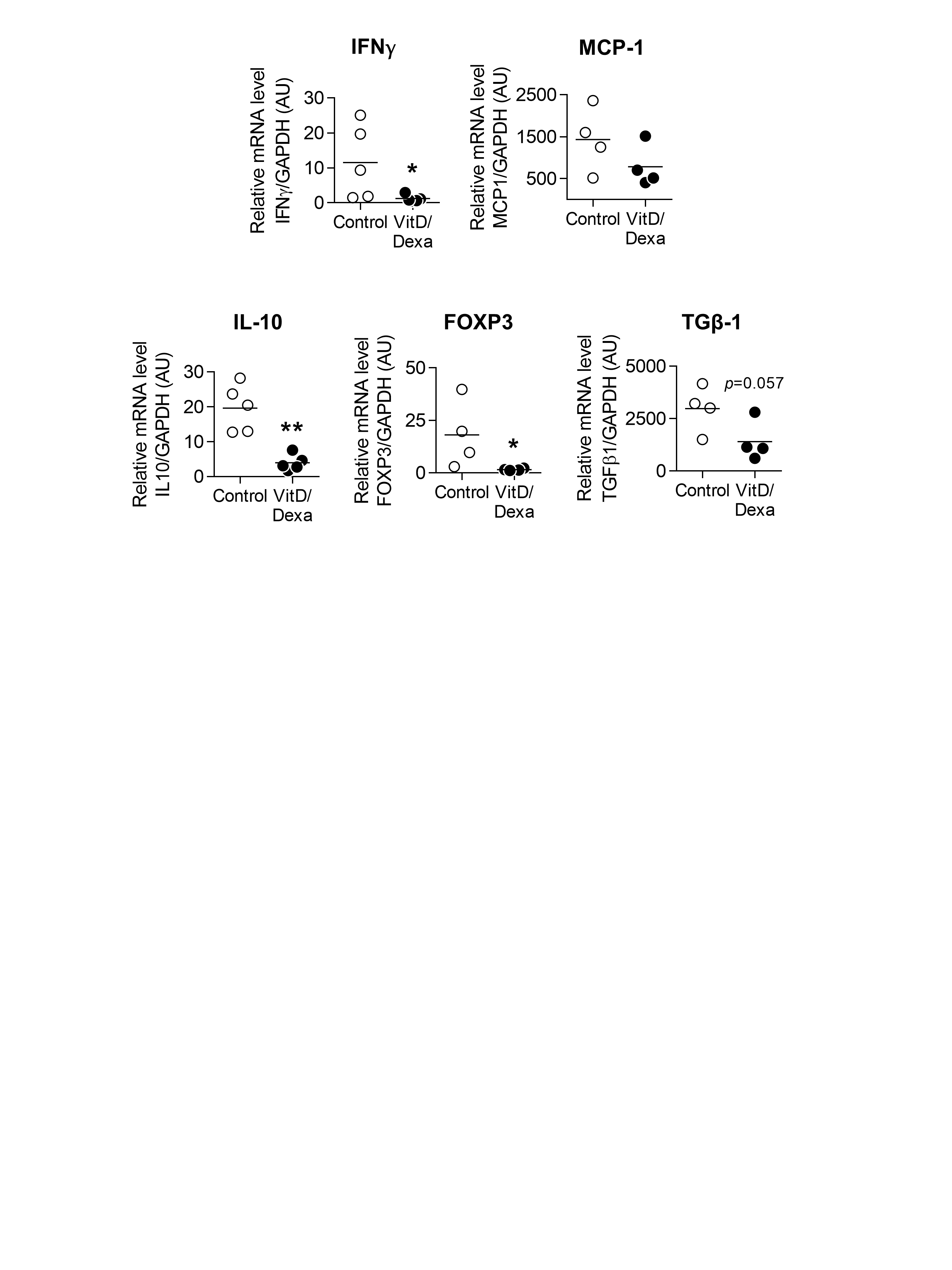
**

**Supplementary Figure S9. mRNA levels of cytokines and FOXP3 in abdominal aortas.** Total mRNA from abdominal aortas of VitD/Dexa- or vehicle-treated mice was obtained to quantify the transcript levels of the indicated marker by quantitative real time PCR. mRNA level per mouse is expressed as arbitrary units (AU) and bars represent the mean from 4-5 mice/group. *P<0.05, **P<0.01 (Mann Whitney test).

# Supplementary Methods

**Mice and Reagents**. Wild-type C57BL/6 and BALB/c mice (Charles River, Portage, MI, USA) and apolipoprotein E-deficient (ApoE^-/-^) mice (B6.129P2-Apoetm1Unc/J, The Jackson Laboratory, Bar Harbor, ME, USA) were bred and maintained at the Animal Facility of the “Sede de Investigación Universitaria, Universidad de Antioquia”, under specific pathogen-free conditions. Gender- and age-matched mice were used in all experiments for all groups. All experiments were approved by the institutional ethical committee (Comité Institucional para el Uso y Cuidado de los Animales de Experimentación, CICUAL) and performed following the relevant local guidelines and regulations. Mice were maintained on a standard chow diet (LabDiet, Richmond, IN, USA) or a high-fat diet (HFD; Harlan Teklad 88137, 42% of kcal from fat, 0.2% cholesterol) for the indicated periods. Vitamin D3 (VitD; ALX-460-026) and Dexamethasone (Dexa; BML-EI126) were purchased from Enzo (Enzo Life Science International, Inc, USA), and stock solutions (20 mg/ml) were prepared in Dimethyl sulfoxide (DMSO; Sigma, D-2650) and stored at -80°C. The CpG oligodeoxynucleotide (ODN) 1826 (5'-TCCATGACGTTCCTGACGTT-3') was synthesized by Integrated DNA Technologies (Coralville, IA, USA) and was phosphorothioate-modified. Freund's complete adjuvant (CFA; F-5881) and ovalbumin (OVA; A-5503) were obtained from Sigma-Aldrich (USA). Human native and oxidized low-density lipoproteins (nLDL and oxLDL, respectively) were prepared and characterized, as previously described (2).

**Treatment of mice and sample obtention.** In pilot screening experiments we tested the forkhead box P3 (FOXP3) T regulatory (Treg)-inducing capacity of a collection of commercially available synthetic or natural small immunosuppressive, anti-inflammatory and/or antioxidant molecules, with documented tolerogenic dendritic cell (tolDC)/Treg-inducing activity. These included Dexa, Rapamycin (Rapa), Acetylsalicylic Acid (ASA), Quercetin (Querc), Curcumin (Curc), *N*-acetylcysteine (NAC), Vitamin C (VitC), Vitamin E (VitE) and VitD (3–7). This preliminary screening was performed using subcutaneous (sc), low-dose, and repetitive injections, since preliminary evidence suggest this as a convenient and effective via/scheme for Treg induction (8) with potential for human translation. Pilot experiments revealed the combination of VitD/Dexa as the formulation yielding the highest increase in the frequency of splenic CD4^+^ CD25^+^ FOXP3^+^ Tregs in wild type mice, under conditions in which the atherosclerosis-related antigen LDL was co-administered or not (see **Supplementary Figure S1** for details on the scheme and results). These results were concordant with the well-established synergy between the vitamin D receptor (VDR) and the glucocorticoid receptor (GR) as anti-inflammatory and Treg-inducing adjuvants (9–13) and prompted us to further investigate the formulation VitD/Dexa in the context of dyslipidemia and atherosclerosis. For this, male 8-12 weeks old ApoE^-/-^ were injected s.c. into the footpad on days 0, 4 and 7 with the combination of VitD and Dexa (10 µg each, in a final volume of 50 µl, diluted in PBS from stock solutions). Vehicle control mice were treated with 2% DMSO diluted in PBS (Gibco-Live Technologies, USA). This scheme was repeated seven times with weekly intervals. Mice were fed a standard chow diet during the first 2 rounds of treatment (until day 21), and then shifted to HFD for the rest of the experiment (12 weeks), as shown in **Figure 1**. After this, animals were sacrificed to obtain the following samples: i) Hearts-aortas were collected from PBS-perfused mice, immersed in 4% paraformaldehyde (PFA; JT Baker, USA) for 48 h and then in 30% sucrose (Sigma-Aldrich, USA) for 24h at 4°C. Subsequently, organs were embedded in Shandon Cryomatrix resin (Thermo Scientific) and kept at -20°C until processing for atherosclerotic lesion size determination and immunohistochemistry (IHC). ii) Spleens (SP) and popliteal lymph nodes (PLN) were removed and placed in RPMI 1640 (GlutaMAX, Gibco, USA). Spleens were perfused with RPMI 1640. Organs were then mechanically dissociated by pressing through a 74-μm mesh filter (Spectra Mesh® Woven Filters, USA) with the plunger of a syringe. Spleen dissociation was followed by red blood cell lysis with 1x RBC lysis buffer (eBioscience, USA). The processed single-cell suspensions were resuspended in RPMI 1640 (GlutaMAX) supplemented with 10% fetal bovine serum (FBS, heat-inactivated) and Penicillin/Streptomycin (100 U/ml, 100 µg/ml, respectively; Gibco, USA), further referred to as supplemented cell culture medium. Cell suspensions were then used to assess the induction of tolerogenic (IL-10-producing) antigen-presenting cells (APCs), Tregs (IL-10-producing CD4^+^ T cells, called here Tr1-like Tregs and forkhead box P3^+^ (FOXP3^+^) CD4^+^ T cells, called here FOXP3^+^ Tregs), Th1 cells (IFNγ -producing CD4^+^ T cells), and myeloid-derived suppressor cells (MDSCs) by flow cytometry. Mediastinal lymph nodes (MLN) were also obtained and processed similarly but given the poor yield and cell viability typically observed in this organ, cell suspensions were used only for Treg and MDSC staining in some experiments. Finally, iii) Blood samples were obtained by cardiac puncture, processed to obtain serum and stored at -80°C until further use for biochemical analysis. Two additional atheroprotection pilot experiments were finally performed: 1) 8-12 weeks old male ApoE^-/-^ mice were treated sc with VitD (10 μg) or vehicle and fed a HFD, as shown in **Figure 1**. 2) A similar protocol was used in an independent experiment in which groups of 11-17 weeks old female ApoE^-/-^ mice were treated sc with Dexa (10 μg), VitD/Dexa (10 μg/10 μg) or vehicle. After sacrifice, hearts-aortas were obtained and processed for atherosclerotic plaque size determination.

**Flow cytometry**. For intracellular cytokine staining, cells (1×10^6^ cells, in 1 ml; 12 x 75 mm BD tubes) were stimulated in supplemented cell culture medium at 37°C for 4 h with 5 ng/ml PMA (Sigma, P-8139) and 500 ng/ml ionomycin (Sigma, I-0634), in the presence of either Monensin (2 µM; eBioscience) for IL-10 detection or Brefeldin A (1 µl/ml; BD GolgiPlug) for IFNγ detection. Following incubation, cells were harvested, washed with cold PBS and incubated 15 min at 4°C with anti-mouse CD16/CD32 (2.4G2, BD FcBlock) mAb in the presence of a fixable viability staining (Live/dead fixable stain kit, Invitrogen). Cells were subsequently suspended 10 min in blocking buffer (10% FBS, 0.1% BSA and 0.01% sodium azide in PBS) and stained in FACS buffer (3% FBS and 0.09% sodium azide in PBS) for 20 min at 4 °C with the appropriate combinations of the following monoclonal antibodies (mAbs, from BD Pharmingen): CD3 Molecular Complex (17A2), CD3e (145-2C11), CD4 (GK1.5), CD4 (RM4-5), CD8a (53-6.7), CD25 (PC61), CD19 (1D3), I-A(b) (AF6-120.1), CD11c (HL3), CD40 (HM40-3), NK-1.1 (PK136). After staining of surface markers, cells were fixed in 2% PFA and permeabilized in PERM buffer (0.1% Saponin, 10% FBS, 0.1% BSA, 1mM de CaCl_2_, 1mM MgSO_4_·7H_2_O, 40mM HEPES and 0.09% sodium azide in PBS), followed by staining with anti IL-10 (JES5-16E3) or anti IFNγ (XMG1.2) mAbs (all from BD). For FOXP3 staining, cells were kept unstimulated, and an anti FOXP3 (MF23) mAb or a Rat IgG2b κ Isotype Control (A95-1) were used according to manufacturer’s instructions (FOXP3 staining, BD). For myeloid-derived suppressor cell (MDCS) staining, cells were kept unfixed and anti CD11b (M1/70), CD11c (HL3), and Gr1 (RB6-8C5) mAbs were used according to manufacturer’s instructions. Acquisitions were performed on a FACSCanto-II or an Accuri C6 (BD Biosciences) flow cytometers and data analyzed using FlowJo software (Tree Star). Acquisitions were at least 100.000 live cells for SP and PLN, and 15.000 live cells for MLN. Gates were set based on unstimulated samples and Fluorescence Minus One (FMO) controls. In general, no major changes in the frequency or absolute numbers of parent populations of APCs or T cells were observed in cells from VitD/Dexa-treated mice as compared to vehicle-treated mice.

***In vitro* cytokine secretion assays.** Splenic single-cell suspensions from VitD/Dexa- and vehicle-treated ApoE^-/-^ mice were resuspended in supplemented cell culture medium at a density of 1.5 × 10^6^ cells/ml. A mitogenic polyclonal stimulation of T cells by Concanavalin A (ConA; final concentrations 0, 0.1, 0.3 or 1 µg/ml) (Sigma, C-5275) was performed during 48h at 37°C, 5% CO_2_. After culture, IFNγ and IL-10 production was analyzed by ELISA (Mouse IFNγ ELISA set, assay range 3.1 - 200 pg/ml; Mouse IL-10 ELISA set, assay range 31.3 - 2000 pg/ml; BD OptEIA).

**Atherosclerotic lesion measurements, immunohistochemistry and qPCR.** Hearts-aortas were processed to assess the size of atherosclerotic lesions, as described previously (2). Briefly, frozen hearts were cut into 6-7 µm thickness sections with a cryostat (Leica, Germany), from the start of the aortic sinus to the start of the ascending aortic arch, and mounted on charged glass slides (Superfrost Plus, Thermo Scientific). Sections were stained with conventional Hematoxylin/Eosin (H/E) or Oil Red O to assess lesion size and lipid deposition, respectively. Slides were also used to determine macrophage and T cell infiltration by IHC. For this, cryosections were acetone-fixed and subjected to antigen retrieval by steaming the slides in 10 mM Tris-HCl buffer (pH 6.0) for 5 min and cooling at room temperature for 10 min. Endogenous peroxidase was blocked by incubation in methanol with 3% H_2_O_2_ for 15 min at RT. Sections were incubated with a blocking solution (1% BSA, 0.3% Triton X-100 in PBS) 60 min before incubation with anti-mouse CD3 (KT3; 1:25) or anti Macrophages/Monocytes (MOMA-2; 1:20) antibodies (AbD Serotec) at 4°C, overnight. For some experiments, macrophage infiltration was quantified by staining the CD68 marker (FA-11; 1:50; AbD Serotec). After washing in PBS, sections were incubated with horseradish peroxidase-conjugated secondary antibody (goat anti-rat IgG: HRP, mouse adsorbed; 1:50; AbD Serotec) for 1h at RT, followed by 3,3′-diaminobenzidine (AbD Serotec) for visualization. Finally, the sections were counterstained with hematoxylin. Stained slides were observed under an E200 microscope (Nikon), and images were captured using the DS-Fi1 camera, at a 40X magnification. Lesion size was defined by the area between the internal elastic lamina and the luminal boundary. Lesion size, as well as lipid deposition (Oil Red O^+^), macrophage infiltration (MOMA-2^+^), and T cell infiltration (CD3^+^) areas, were calculated using the ImageJ software. Results were reported as the mean area in μm^2^ out of 7-9 sections per mouse. Abdominal aortas were preserved in RNAlater (Qiagen Inc., Germantown, MD, USA) and stored at −80°C until use. Total RNA was isolated using the RNeasy™ Mini Kit (Qiagen, Germany). cDNA was synthesized from 100 ng of RNA using a Revert Aid H Minus First Strand cDNA synthesis kit (Thermo Scientific Inc., Waltham, MA). MCP-1, IFNγ, IL-10, TGβ-1, and FOXP3 were amplified by quantitative PCR (qPCR) using Applied Biosystems™ TaqMan® Gene Expression Assays (Foster, CA, USA) on a LightCycler™ 96 (Roche, Penzberg, Germany). Relative mRNA levels were calculated for each animal using the ΔCt method and normalized to Glyceraldehyde-3-Phosphate Dehydrogenase (*GAPDH*) as a reference gene.

**Serum biochemical analysis.** Serum cholesterol and triglycerides were determined with dry chemistry methods (Johnson & Johnson). Quantification of the circulating levels of the lipid peroxidation product malondialdehyde (MDA) was performed by using the thiobarbituric acid-reactive species method and a standard curve, as described previously (2). Calcium levels were determined enzymatically by using an automated chemistry analyzer (Biosystems A-15). Serum glucose was measured by glucose oxidase-peroxidase method using the reagent kit purchased from BioSystems (Spain).

***Ex vivo* suppression assay.** To test *in vitro* the suppressive activity of CD4^+^ T cells after *in vivo* VitD/Dexa treatment, chow diet-fed C57BL/6 mice were injected into the footpad with VitD/Dexa (10 µg each) or vehicle on days 0, 2, 4 and 6. This regimen was repeated four times with weekly intervals. Mice were then sacrificed, and the spleens removed to isolate splenic CD4^+^ T cells by FACS-sorting using a MoFlo XDP cell sorter (Beckman-Coulter). In parallel, chow diet-fed C57BL/6 mice were immunized into the footpad with oxLDL (12.5 µg), CpG (20 µg) and CFA (20µl) prepared in PBS (on day 0) to generate oxLDL-specific effector Th1 cells. Mice were boosted on days 7, and 14 with oxLDL (12.5 µg) and CpG (20 µg) dissolved in PBS, to be finally sacrificed (on day 25) to obtain the spleens. FACS-sorted CD4^+^ T cell populations from VitD/Dexa-treated or control mice were co-cultured with total SP cells from oxLDL-vaccinated mice (containing IFNγ-producing effector T cells; Teff), in the presence of LDL (10 µg/ml) or the irrelevant antigen (OVA, 60 µg/ml) for 72 h. The suppressive activity was then determined by the amount of IFNγ released to the medium at different Teff:TCD4^+^ ratios (30:1, 10:1, 3:1, 1:1) by ELISA.

**Immunosuppressive effect of VitD/Dexa on a chronic persistent intracellular infection**. The virulent *Leishmania panamensis* isolate (MHOM/CO/93/UA946) was maintained by passage in BALB/c mice. Promastigotes were grown *in vitro* in Schneider’s insect medium (SIM; Sigma) supplemented with 10% FBS (heat-inactivated), penicillin/streptomycin (100 U/ml/100 µg/ml). The infectivity of this inoculum was confirmed by infecting BALB/c mice in the ear dermis with 1x10^5^ stationary-phase *L. panamensis* promastigotes, and by monitoring the development of cutaneous ulcers up to for 8 weeks postinfection. Wild type C57BL/6 mice were also infected with a similar inoculum. The course of infection was monitored weekly by measuring the lesion size. Once C57BL/6 mice were completely cured and entered the latent phase of infection (around 7-8 weeks postinfection), were randomly divided into two groups and injected into the footpad with VitD/Dexa or vehicle, using the same scheme and dose that resulted in atheroprotection (**Figure 1**). An overall assessment of the health of mice was made throughout the experiment paying particular attention to the infected ear. Animal’s overall appearance, activity level, hydration status, body condition, and the presence of abnormalities, was evaluated daily. Body weight was assessed every week. For determination of the number of viable parasites in the infected ear after treatment, a limiting dilution assay was used. Single-cell suspensions were prepared and serially diluted (1:3 dilutions) across a 96-well flat-bottom tissue culture plate in Complete SIM, in quadruplicate, and incubated at 26 °C for 30 days. At this time, the greatest dilution yielding viable parasite growth was recorded for each mouse to calculate the number of viable parasites per organ.

**Statistical analyses.** Statistical analysis was performed using GraphPad Prism 6.0 software. Data were tested for normality using the D'Agostino-Pearson normality test. For comparison of unmatched two groups, unpaired Student’s t-test or Mann-Whitney U-test was used. To take account of multiplicity in group comparisons, one- or two-way ANOVA followed by Bonferroni’s posttest was conducted. Equality of variances was evaluated using the Levene test. Results are presented in figures as the mean, the mean±SEM, the mean±SD or the geometric mean±95%IC. P<0.05 was considered significant (*P<0.05; **P<0.01; ***P<0.001; ****P<0.0001).

**References**

1. Younos I, Donkor M, Hoke T, Dafferner A, Samson H, Westphal S, Talmadge J. Tumor- and organ-dependent infiltration by myeloid-derived suppressor cells. *Int Immunopharmacol* (2011) **11**:816–826. doi:10.1016/j.intimp.2011.02.021

2. Lara-Guzman OJ, Tabares-Guevara JH, Leon-Varela YM, Álvarez RM, Roldan M, Sierra JA, Londoño-Londoño JA, Ramirez-Pineda JR. Proatherogenic Macrophage Activities Are Targeted by the Flavonoid Quercetin. *J Pharmacol Exp Ther* (2012) **343**:296–306. doi:10.1124/jpet.112.196147

3. Tan PH, Sagoo P, Chan C, Yates JB, Campbell J, Beutelspacher SC, Foxwell BMJ, Lombardi G, George AJT. Inhibition of NF-κB and Oxidative Pathways in Human Dendritic Cells by Antioxidative Vitamins Generates Regulatory T Cells. *J Immunol* (2005) **174**:7633–7644. doi:10.4049/jimmunol.174.12.7633

4. Huang R-Y, Yu Y-L, Cheng W-C, OuYang C-N, Fu E, Chu C-L. Immunosuppressive Effect of Quercetin on Dendritic Cell Activation and Function. *J Immunol* (2010) **184**:6815–6821. doi:10.4049/jimmunol.0903991

5. Hackstein H, Thomson AW. Dendritic cells: emerging pharmacological targets of immunosuppressive drugs. *Nat Rev Immunol* (2004) **4**:24–35. doi:10.1038/nri1256

6. Cong Y, Wang L, Konrad A, Schoeb T, Elson CO. Curcumin induces the tolerogenic dendritic cell that promotes differentiation of intestine-protective regulatory T cells. *Eur J Immunol* (2009) **39**:3134–3146. doi:10.1002/eji.200939052

7. Buckland M, Jago C, Fazekesova H, George A, Lechler R, Lombardi G. Aspirin modified dendritic cells are potent inducers of allo-specific regulatory T-cells. *Int Immunopharmacol* (2006) **6**:1895–1901. doi:10.1016/j.intimp.2006.07.008

8. Kang Y, Xu L, Wang B, Chen A, Zheng G. Cutting Edge: Immunosuppressant as Adjuvant for Tolerogenic Immunization. *J Immunol* (2008) **180**:5172–5176. doi:10.4049/jimmunol.180.8.5172

9. Nikolic T, Roep BO. Regulatory Multitasking of Tolerogenic Dendritic Cells – Lessons Taken from Vitamin D3-Treated Tolerogenic Dendritic Cells. *Front Immunol* (2013) **4**:113. doi:10.3389/fimmu.2013.00113

10. Kalra N, Ishmael FT. Cross-talk between vitamin D, estrogen and corticosteroids in glucocorticoid resistant asthma. *OA Inflamm* (2014) **2**:2.

11. Wöbke TK, Sorg BL, Steinhilber D. Vitamin D in inflammatory diseases. *Front Physiol* (2014) **5**:244. doi:10.3389/fphys.2014.00244

12. Litonjua AA. Vitamin D and corticosteroids in asthma: synergy, interaction and potential therapeutic effects. *Expert Rev Respir Med* (2013) **7**:101–104. doi:10.1586/ers.12.85

13. Gordon JR, Ma Y, Churchman L, Gordon SA, Dawicki W. Regulatory Dendritic Cells for Immunotherapy in Immunologic Diseases. *Front Immunol* (2014) **5**:7. doi:10.3389/fimmu.2014.00007
